# Wide‐/Narrow‐Bandgap Heterojunction for High Performance Differential Photodetector with Tunable Response

**DOI:** 10.1002/advs.202504872

**Published:** 2025-07-29

**Authors:** Ziyang Ren, Haimin Zhu, Weien Lai, Yihui Zhai, Mengjuan Liu, Yu Zhu, Hanlun Xu, Nasir Ali, Ning Dai, Jiaqi Zhu, Sihan Zhao, Huizhen Wu

**Affiliations:** ^1^ School of Physics Zhejiang University Hangzhou Zhejiang 310027 China; ^2^ Zhejiang Key Laboratory of Micro‐nano Quantum Chips and Quantum Control Zhejiang University Hangzhou Zhejiang 310027 China; ^3^ State Key Laboratory of Silicon and Advanced Semiconductor Materials Zhejiang University Hangzhou Zhejiang 310027 China; ^4^ National Engineering Laboratory of Special Display Technology Hefei University of Technology Hefei Anhui 230009 China; ^5^ School of Instrument Science and Opto‐electronics Engineering Academy of OptoElectronic Technology Hefei University of Technology Hefei Anhui 230009 China; ^6^ Hangzhou Institute for Advanced Study University of Chinese Academy of Sciences Hangzhou Zhejiang 310024 China; ^7^ Intelligent Manufacturing Computing Research Center Zhejiang Lab Hangzhou Zhejiang 310027 China

**Keywords:** bandwidth, differential photodetector, heterojunction, transient photoresponse, wide‐bandgap semiconductor

## Abstract

Amid the rapid advancement in modern photonics and artificial intelligence, optoelectronic devices with enhanced functionalities and high performance hold great promise for complex photonic integrated circuits. Herein, a novel tunable differential photodetector (DPD) is developed based on a wide‐/narrow‐bandgap semiconductor heterojunction, featuring a large conduction band offset at the heterojunction interface. Benefiting from the unique band alignment at the heterojunction interface, this device exhibits interesting characteristics:1) It offers two tunable operational modes, freely switchable between “differential mode” and “normal mode” only by adjusting the bias voltages; 2) While operating in “differential mode” under zero bias, the DPD exhibits high detectivity (4.5×10^11^ Jones) and broad bandwidth of ≈1 MHz under 1550 nm laser at room temperature; 3) Due to the narrow bandgap of PbSe, the device operates at longer wavelengths than reported to date. An equivalent circuit model is proposed to elucidate the working mechanism that is experimentally observed. The practical applications of the DPD in the event‐based imaging of a moving flame and encrypted communication are further demonstrated. The work establishes a novel approach for optoelectronic devices toward multifunctional integrated photonics applications.

## Graphical abstract

A differential photodetector is developed by applying the unique characteristics of a wide‐/narrow‐bandgap semiconductor heterojunction. The mechanism based on band alignment enables high detectivity and high bandwidth with tunable photoresponse. Applications of the DPD are demonstrated in the event‐based imaging of a moving flame and encrypted communication, showing great potential for multifunctional integrated photonics development.

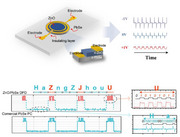

## 1 Introduction

In the era of information, traditional electronic computers face significant challenges in terms of time and power consumption when processing a massive amount of data. However, as Moore's Law begins to falter, the development of conventional electronic circuits is facing increasing limitations. One factor contributing to this is the limitation of transistor miniaturization caused by quantum tunnelling effects.^[^ ^1^ ^]^ To address this issue, a variety of platforms for information processing are being explored, including optical computing,^[^ ^2^, ^3^ ^]^ quantum computing,^[^ ^4^ ^]^ and molecular computing.^[^ ^5^ ^]^ Compared with conventional electronic systems, optical computing is inherently advantageous in terms of high speed and robustness to electromagnetic interference.^[^ ^6^ ^]^ These features make photonic integrated circuits (PICs) suitable for a wide range of applications, including classical and quantum information processing, neuromorphic computing, and artificial intelligence.^[^ ^7^, ^8^ ^]^ However, several challenges are still hindering the development of PICs. PICs require the integration of various components (photodetectors, lasers, optical waveguides, etc.) onto a single chip.^[^ ^9^ ^]^ One major challenge emerges as the diversity and number of components grow and the differences in materials and fabrication processes make integration increasingly complex,^[^ ^10^, ^11^, ^12^ ^]^ thus hindering the realization of fully integrated photonic circuits.^[^ ^6^ ^]^ Efficient power management remains another major challenge for the development of PICs. Despite the advantages of low energy consumption in PICs, maximizing energy efficiency while effectively modulating and detecting optical signals remains a significant issue to be addressed.^[^ ^13^ ^]^

As a bridge between light and electricity, photodetectors are essential components of PICs. With the rapid development of photonic integration, an increasing number of photodetectors with multiple functions have emerged, such as detectors that simultaneously perform optical demultiplexing and detection.^[^ ^14^ ^]^ These photodetectors could offer promising solutions to resolve the challenges encountered by PICs through significantly reducing the complexity and power consumption. Owing to their unique optoelectronic properties and high performance, heterojunctions based on wide‐ and narrow‐bandgap semiconductor materials hold significant potential for applications in PICs. The integration of wide‐ and narrow‐bandgap semiconductors through forming heterojunctions has resulted in significant performance enhancements, such as precise control over electronic, optical, and thermal characteristics^[^ ^15^, ^16^ ^]^ and carrier confinement.^[^ ^17^, ^18^ ^]^ Additionally, devices with high‐frequency and high‐power performance^[^ ^19^ ^]^ along with high sensitivity and enhanced thermal stability have been realized.^[^ ^20^, ^21^, ^22^ ^]^ In optoelectronic devices, narrow‐bandgap semiconductors can enhance the device's broadband optical absorption, while wide‐bandgap semiconductors can effectively suppress the dark current and enhance the device's stability. Currently, several new functional optoelectronic devices based on wide‐/narrow‐bandgap semiconductor heterojunctions have been reported, such as phototransistor,^[^ ^23^ ^]^ dual‐wavelength demultiplexer,^[^ ^14^ ^]^ and programmable optoelectronic synaptic transistor.^[^ ^24^ ^]^ These developments hold great promise for future applications in opto‐logical systems and neuromorphic computing.^[^ ^24^, ^25^ ^]^ Among them, differential photodetector (DPD), a transient‐type photodetector^[^ ^26^, ^27^, ^28^, ^29^ ^]^ that is capable of detecting and modulating optical signals in situ, is particularly attractive for applications in PICs and encrypted communication. However, many of them cannot simultaneously achieve both high detectivity and broad bandwidth.^[^ ^28^, ^30^, ^31^ ^]^

In this work, we have designed a novel wide‐/narrow‐bandgap heterojunction DPD composed of wide‐bandgap ZnO and narrow‐bandgap PbSe forming a heterojunction interface. A large conduction band offset at the ZnO/PbSe heterojunction interface was observed by *C‐V* characterization. We successfully fabricated ZnO/PbSe heterojunction DPDs with different pixel sizes, and the performance of the devices was thoroughly investigated. Unlike traditional ZnO UV photodetectors, which solely rely on the photosensitive property of ZnO,^[^ ^32^ ^]^ the dielectric property of wide‐gap ZnO in our detector realizes new functionalities in photodetection. Compared with previously reported DPDs using an insulator layer, our DPD has two tunable work modes, making it promising in encrypted communication and tuning between the two operation modes of event cameras. Furthermore, the device features with broad bandwidth and high detectivity under zero bias. This work provides a new strategy for developing multifunctional optoelectronic devices and paves the way for enhancing the DPDs’ performance through a wide range of wide‐/narrow‐bandgap heterostructures.

## 2 Results and Discussion

**Figure 1:**
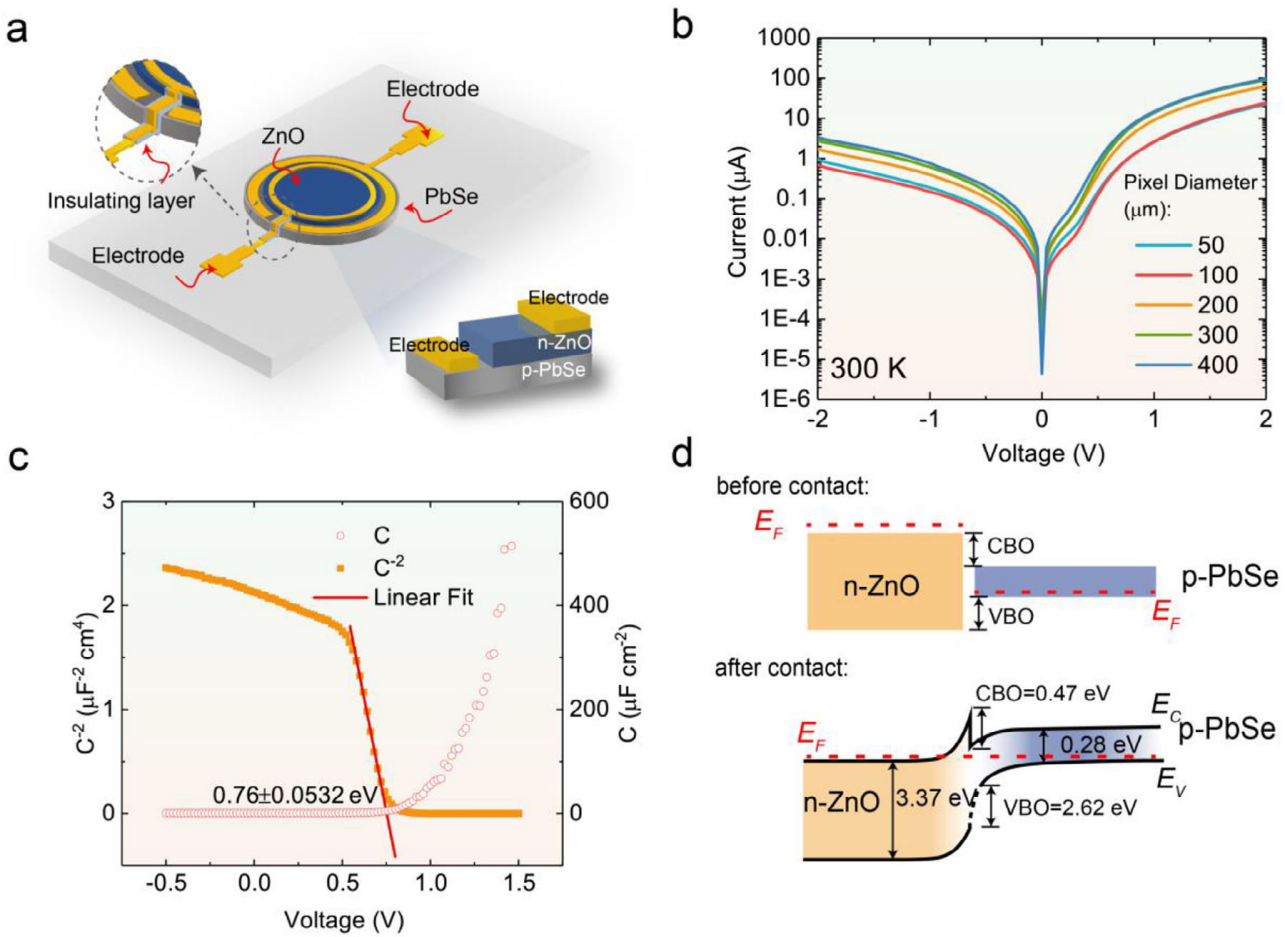
a) A schematic of the device structure. The inset shows a cross‐sectional view of the device. b) The measured *I‐V* curves for ZnO(100 nm)/PbSe(1 µm) DPDs with different pixel sizes under dark conditions. c) The *C‐V* (red circles) and *C^−2^‐V* (orange squares) curves of the device, with the red solid line denoting the fitted data. The intercept between the fitted data (red line) and the horizontal axis is 0.76 ± 0.05 V (statistical results for five samples). The frequency used for the *C‐V* measurement is 1 kHz, and the amplitude of the AC signal is 10 mV. d) Band alignment of the ZnO/PbSe heterojunction before and after contact. The band offsets at the interface are obtained from the *C^−2^‐V* data shown in (c).

**Figure** **1**a shows the device structure of the ZnO/PbSe heterojunction DPD, where the top electrode is insulated from the sidewall of the PbSe layer through an insulating layer. The surface morphology of PbSe and the detailed device structure can be seen in the SEM image and optical microscopic image shown in Figure S2a,b (Supporting Information). Figure 1b shows the *I‐V* curves of ZnO(100 nm)/PbSe(1 µm) heterojunctions with different pixel sizes. The trends of the forward current could be analyzed using the traditional diode theory:^[^ ^33^ ^]^ *I*∝exp (*qV*/*nk*_0_ *T*) − 1, where *q*, *k_0_*, and *T* represent elementary charge, Boltzmann constant, and temperature, respectively. The fitted value of n is ≈3.11, which is bigger than the ideal factor of a traditional diode.^[^ ^33^ ^]^ This result can be attributed to the defects at the ZnO/PbSe interface because of polycrystallinity and lattice mismatch, which can trap a portion of carriers.^[^ ^33^, ^34^ ^]^ Similar *I‐V* curves of ZnO(200 nm)/PbSe(1 µm) and ZnO(400 nm)/PbSe(1 µm) heterojunctions are provided in Figure S2 (Supporting Information). Additionally, Figure 1c shows the *C‐V* and *C^−2^‐V* curves of the heterojunction. The conduction band offset and valence band offset shown in Figure 1d are derived from the *C‐V* data in Figure 1c. We obtain a barrier height of 0.76 ± 0.053 eV through linear fitting and statistical analysis of five samples of ZnO(100 nm)/PbSe(1 µm) heterojunctions. The conduction band offset and valence band offset are concluded to be 0.47 and 2.62 eV, respectively, which is close to those obtained from synchrotron radiation photoelectron spectroscopy.^[^ ^35^ ^]^ The calculation details are provided in the Supporting Information.

**Figure 2:**
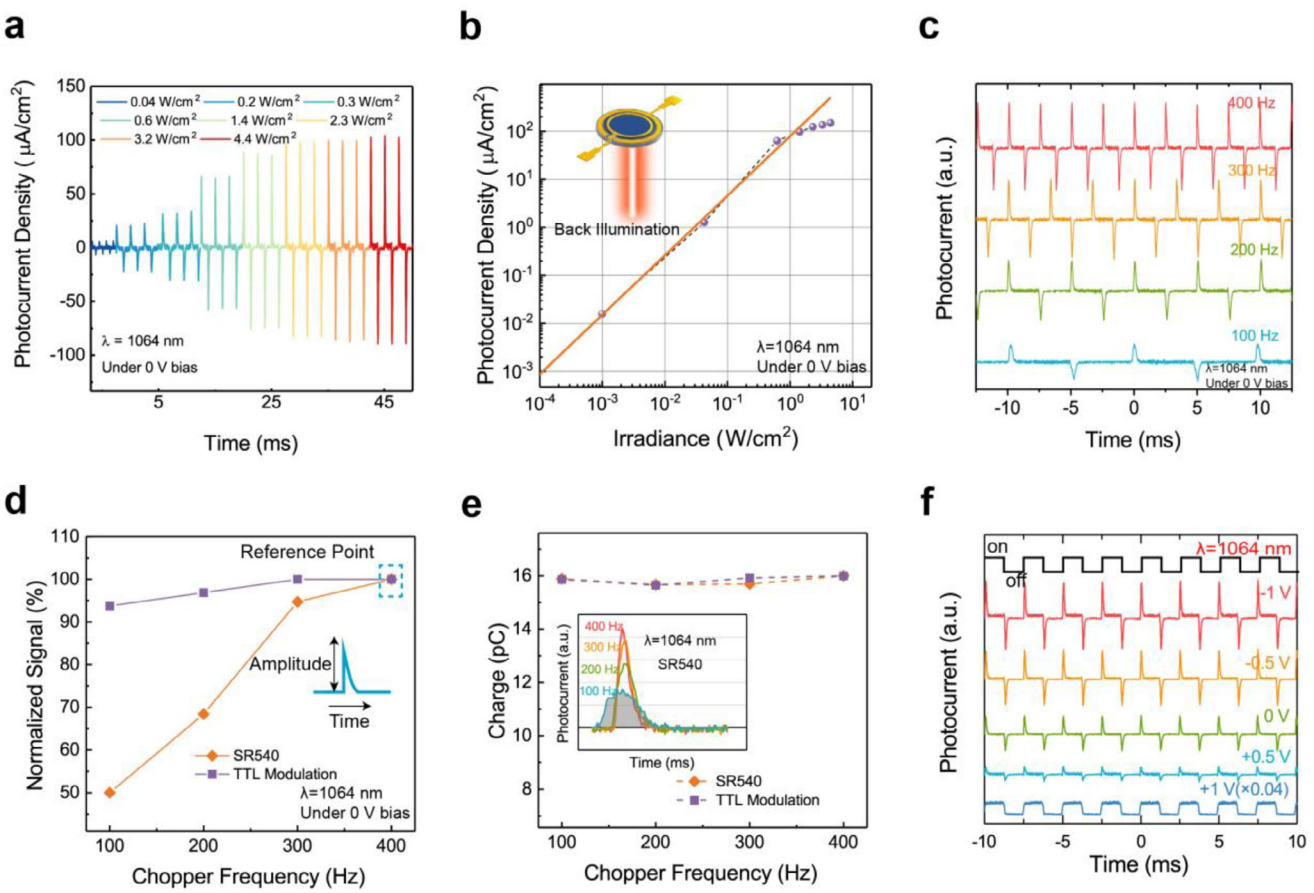
a) The output waveforms of the device under different laser power densities, with a laser wavelength of 1064 nm and pulse frequency of 400 Hz. b) Measured photocurrent density as a function of laser irradiance. The inset schematically illustrates light incidence from the PbSe side. c) The output waveforms of the device with varying pulse frequencies from 100 Hz to 400 Hz, where the laser beam was modulated by using an optical chopper (SR540). The waveforms are measured under zero bias voltage. d) The frequency‐dependent signal amplitude under two different methods (transistor‐transistor‐logic (TTL) and optical chopper) for light‐switching. The data is normalized by the ratio relative to the signal at 400 Hz, respectively. e) The charge under two different methods for light switching (The inset shows detailed waveforms under different frequencies of chopper). f) The output waveforms of the device under different bias voltages were measured at a pulse frequency of 400 Hz.

We measured the dynamic photocurrent response of the device under 1064 nm laser illumination, where the laser was incident from the PbSe side and switched on/off using two methods: an optical chopper and TTL modulation. All the waveforms shown in **Figure** **2** are from optical chopper modulation, while the results of TTL modulation are shown in Figure S3 (Supporting Information). Figure 2a shows the I‐t curves under different light power densities, with different power levels marked in different colors. As shown in Figure 2, under 1064 nm laser illumination, the device does not respond to constant light but instead outputs a pulse signal with polarity when the light intensity changes. Specifically, the device generates a positive or negative pulse when the light is switched on or off, respectively. Figure 2b presents the fitting curve of peak photocurrent as a function of light power, maintaining good linearity at power levels below 1 W cm^−2^. However, when the power density exceeds 1 W cm^−2^, the response gradually deviates from linearity and tends toward saturation. We know that at low light power density, the recombination rate of carriers in the PbSe layer is unaffected by photogenerated carriers.^[^ ^36^ ^]^ However, at high light power density, the recombination rate of carriers in the PbSe layer increases because of high densities of photogenerated electrons and holes, leading to the photocurrent gradually saturating.^[^ ^37^, ^38^, ^39^ ^]^ Figure 2c shows the dynamic photocurrent response curves of the device under different laser pulse frequencies. As the pulse frequency increases from 100 to 400 Hz, the output pulse peak of the device also increases. After normalizing the peak photocurrent under both optical chopper and TTL modulation, we plotted them on the same graph as shown in Figure 2d, where we can clearly observe that the peak photocurrent is more sensitive to the frequency of the optical chopper than to TTL modulation. Because the laser beam has a certain size (beam diameter ≈1 mm^2^), the durations of the rising edge switched by the optical chopper under different pulse frequencies are not constant. We further used a high‐speed pin diode to measure the rising edges of the laser under different pulse frequencies, and the results are shown in Figure S3a (Supporting Information). Based on these results, we can conclude that the steeper the rising edge of the light, the higher the peak current. We further calculated the integrated charge from the I‐t curves. As shown in Figure 2e, the integrated charge does not change with modulation frequency. The mechanism will be discussed in more detail below. Figure 2f presents the I‐t curves of the device under different bias voltages, where the forward and reverse biases are labeled according to the p‐n junction convention (i.e., “+1 V” means ZnO is grounded, and PbSe is connected to a +1 V voltage). It is observed that under reverse bias and small forward bias, the output signal still maintains the characteristic of pulse waveforms. However, when the forward bias on the device exceeds a threshold voltage (≈0.6 V as shown in Figure S4, Supporting Information), the output waveform turns into a square wave, indicating that the device shows normal continuous photoresponse, as shown by the blue curve at the bottom of Figure 2f with a +1 V bias voltage.

**Figure 3:**
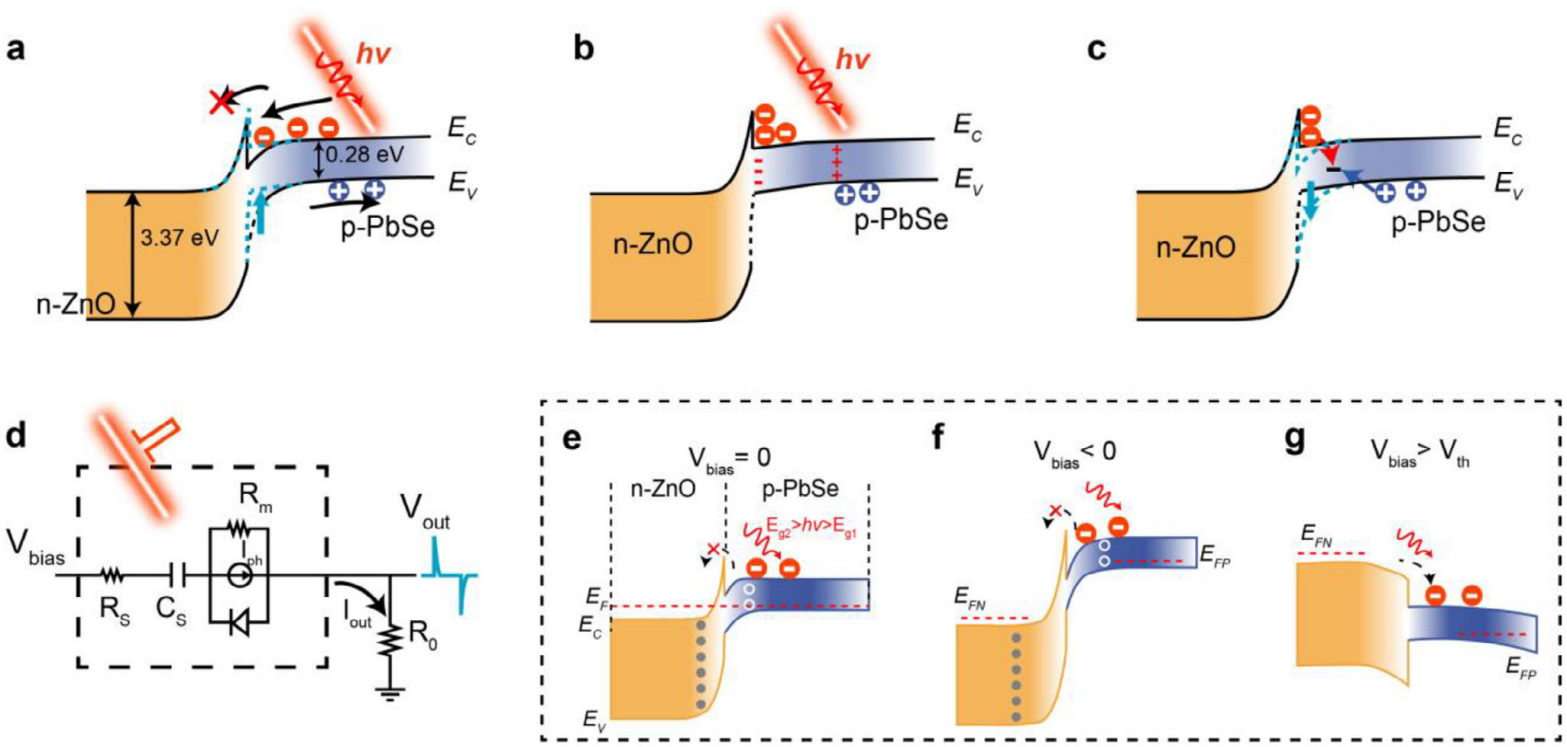
a) The band alignment at the beginning of light illumination. The blue dotted lines represent the band alignment under light illumination. b) The band alignment in steady state under light illumination. c) The corresponding band alignment when the light is turned off. d) The equivalent circuit diagram derived from processes (a) to (c), where the accumulation and recombination of photo‐generated carriers are approximated by a capacitor, and the transient current generated by illumination is represented in the form of a current source. e) Band alignment under zero bias voltage, where the incident photon energy is between the energy gaps of PbSe and ZnO. f) Band bending becomes more pronounced under reverse bias, and g) the band alignment at the interface flattens out under forward bias.

**Figure** **3**a–c shows the energy band alignment in the ZnO/PbSe heterojunction DPD and the distribution of photo‐generated carriers during one cycle of light‐switching under zero bias. Whether illuminated at a wavelength of 1064 or 1550 nm, the photo‐generated carriers are produced only on the PbSe side because of the wide bandgap of ZnO. The absorption spectra of ZnO and PbSe are shown in Figure S5 (Supporting Information). Therefore, throughout the whole process, we focus on the behaviors of non‐equilibrium minority carriers (electrons) on the PbSe side. Due to the conduction band offset, ZnO acts as a dielectric layer to block photo‐generated carriers in PbSe from going into the ZnO side. Meanwhile, the bent energy band of PbSe leads to the accumulation of photo‐generated electrons at the interface. The photo‐generated holes in PbSe are easily collected by the PbSe electrode, resulting in charge displacement within the device, which can be equivalently modeled as a capacitor.^[^ ^40^, ^41^ ^]^ Regardless of reverse or zero bias, the structures of band alignment are similar, which makes the device operate in a “capacitive mode”. Here we discuss the variation of photocurrent during one light‐switching cycle in detail: 1) In the dark, since no photo‐generated carriers are produced, there is no photocurrent in the circuit. 2) As soon as the light is turned on, a large number of photo‐generated carriers are produced, while the capacitor is not yet saturated. Thus, the capacitor is in the charging state. Electrons drift toward the interface under the influence of the electric field in the space charge region. Meanwhile, photo‐generated holes are swept out of the space charge region onto the PbSe electrode, as shown in Figure 3a. This charge movement results in a drift current that quickly rises to its maximum value.^[^ ^14^ ^]^ However, the accumulated negative charges at the interface weaken the built‐in electric field on the PbSe side, as shown in the calculation results in Figure S6 (Supporting Information). And the energy band bending flattens out as shown in Figure 3b, thus hindering the drift of new photo‐generated electrons, causing the current to gradually decrease to zero. Due to charge accumulation, the capacitor is quickly charged and reaches saturation. 3) During the steady‐state illumination stage, because the capacitor was fully charged, it behaves like an open circuit, with no current flowing through the circuit, as shown in Figure 3b. 4) At the moment upon light off, no new photo‐generated carriers are available to supply the interface. The electrons trapped in the interface recombine with holes through the recombination centers in PbSe (the depletion region),^[^ ^30^, ^40^, ^42^ ^]^ as illustrated in Figure 3c. Consequently, a reverse current rapidly rises to its maximum value and then returns to zero as all the photo‐generated electrons are recombined, thus, one cycle is completed. Based on the above theoretical model, we could explain the phenomenon seen in Figure 2d,e: The accumulated charge level at the interface changes with the density of photo‐generated carriers; therefore, it varies with light power density but not with modulation frequency. However, the generation rate of photo‐generated carriers increases as the rising edge of light shortens; thus, the peak current is more sensitive to the frequency of the optical chopper than TTL modulation, and a higher photocurrent is generated at high modulation frequency.

To clearly explain the mechanism of the device operation, we provide the equivalent circuit schematic shown in Figure 3d. In this diagram, *R_S_* is the series resistance of the device, *R_m_* corresponds to the leakage current resistance. *C_S_* is the equivalent capacitance caused by the interface band offset, and *I_ph_* is the photo‐generated current within the device. *R_0_* is the load resistance equivalent in the external circuit, and *I_out_* is the current measured experimentally. Ignoring the effects of series resistance, the following equation can be derived from this model:^[^ ^41^, ^43^ ^,^ ^44^ ^]^

(1)

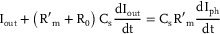

where 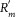 is the parallel resistance of the leakage current resistance and the equivalent resistance of the diode. The solution to Equation 1 is provided in the Supporting Information. It shows that the falling time of the signal is primarily determined by the equivalent capacitance *C_S_* and the load resistance *R_0_*. Reducing the values of these two components helps improve the response speed of the device. We also performed a simulation based on this circuit model, and the results are shown in Figure S7 (Supporting Information). It should be noted that this equivalent capacitance is different from the space‐charge capacitance. The equivalent capacitance arises from the accumulation and recombination of carriers near the interface, while the barrier capacitance comes from the built‐in electric field.

The I‐t curves under different bias voltages in Figure 2f can be illustrated by the energy band alignments in Figure 3e,f. Under reverse bias in Figure 3f, the energy bands are essentially consistent with those under zero bias shown in Figure 3e. The difference is that the energy band bending of PbSe at the interface becomes more pronounced, and the electric field strengthens as shown in Figure 3f. This enhancement increases the drift velocity of electrons, resulting in an increased transient current. In contrast, under small forward bias, energy band bending flattens out and the electric field weakens; thus, the transient current decreases. When the forward bias exceeds a threshold, the energy band changes to the configuration shown in Figure 3g. At this state, the interface loses its charge storage capacity, and the device operating mode shifts from “capacitive” to “normal” mode, showing normal continuous photoresponse characteristics. Therefore, the output waveform excited by rectangular light pulses is also a rectangular one.

**Figure 4:**
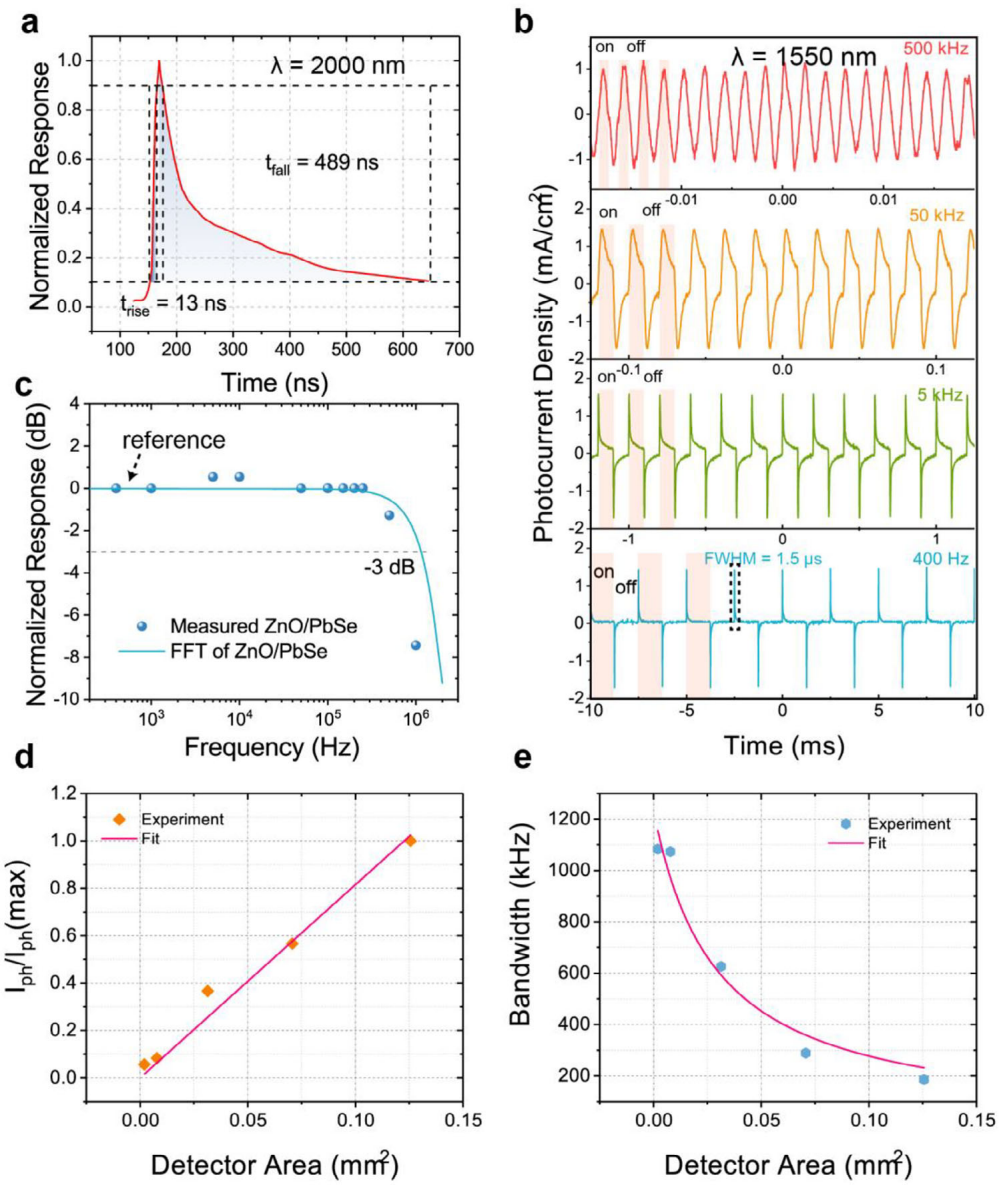
a) The transient photoresponse of the device at room temperature with 2000 nm laser excitation. The rise and fall times of the device are marked. They are defined as the time segment from 10 to 90% of the peak response (or from 90 to 10% during the falling edge), as indicated by the black dashed boxes. b) The output waveforms of the device under 1550 nm DFB semiconductor laser illumination at different modulation frequencies (400 Hz to 500 kHz). The regions with and without light illumination are indicated by the pink and white stripes for the several light switching cycles on the left of each figure panel. c) Bode plot of the device photoresponse. The circles represent the measured photoresponse at different frequencies, and the blue solid line represents the corresponding FFT result obtained from the decay profile shown in Figure 4a. d,e) Normalized photocurrent I_ph_/I_ph_(max) and bandwidth measured at λ = 1310 nm as varying detector areas, where I_ph_(max) is the peak photocurrent of the detectors with a diameter of 400 µm.

To investigate the fast response properties of the device, we used a pulsed laser to measure DPD photoresponse decay. As shown in **Figure** **4**a, the rising time of the device, defined as the time segment from 10 to 90% of the peak response, is 13 ns. Similarly, the falling time, defined as the time segment from 90 to 10% of the peak response, is 489 ns. The falling edge is ≈40 times of magnitude long as the rising edge. In the ZnO/PbSe system, the mechanism is similar to previously published photodetectors based on lead‐salt semiconductors and their heterojunctions,^[^ ^18^, ^36^, ^45^ ^]^ since most holes are swept out of the space charge region, and the probability of recombination with electrons accumulated at the interface is also reduced, thereby extending the time required for decay. Figure 4c shows the response of the device as a function of frequency, which was derived by fast Fourier transform (FFT) calculation of the decay data in Figure 4a. The −3 dB cutoff frequency is ≈1.1 MHz (as marked by the dashed line in Figure 4c). Besides, the RC time constant is 100 ns calculated from the I‐V curves and C‐V curves in Figure 1, which is consistent with −3 dB cutoff frequency.

Figure 4b shows the frequency dependence of the device photoresponse. The schematic of the experiment setup is shown in Figure S8 (Supporting Information). We connected the device to a high‐bandwidth current amplifier (Gain: 10^6^ V A^−1^, Bandwidth: 1.8 MHz) and modulated a 1550 nm laser by TTL square waves with frequencies ranging from 400 Hz to 1 MHz. More detailed waveform curves are shown in Figure S9 (Supporting Information). Similar to the output waveforms under 1064 nm laser illumination, at low frequencies, a positive/negative transient photocurrent is generated upon light on/off, and the two opposite peaks are well balanced. The full‐width‐at‐half‐maximum (FWHM) at low frequency is measured as 1.5 µs, shown in Figure 4b, which is larger than the result in Figure 4a. The 1550 nm laser's pulse width (rising/falling time≈500/800 ns) and pre‐amplifier bandwidth may broaden the FWHM, which is shown in Figure S10 (Supporting Information). As the frequency gradually increases, the transient current does not fully decay to zero, and the recovery process is not complete before the next cycle begins. This results in progressively steady output waveforms like pseudo‐square waves as the frequency increases. Specific waveforms at 250 kHz and explanations can be found in Figure S9 (Supporting Information). The pseudo‐square wave patterns persist from 100 to 500 kHz. Starting from 500 kHz, the waveform changes to a triangular wave, and the amplitude begins to go down, with the final detectable frequency reaching ≈1 MHz, as shown in Figure S9b (Supporting Information). Using the peak current at the low frequency of 400 Hz in Figure 4b as a reference, the directly measured frequency‐dependent photoresponse is plotted by the circles in Figure 4c for comparison. We also measured the peak photocurrent and bandwidth of the detectors with different sizes, as shown in Figure 4d,e. The detailed information can be seen in Figures S11 and S12 (Supporting Information). As the size increases, the peak photocurrent increases linearly, but the bandwidth gradually decreases. The fitted results show that the measured data are in good agreement with the equivalent circuit model (See Equations S9 and S10, Supporting Information).

Based on the theoretical model provided in the Supporting Information, we calculated the figures of merit for the device under 1550 nm laser illumination. The low‐frequency (400 Hz) peak responsivity is ≈0.57 mA W^−1^ (see Figure S13 and Table S1, Supporting Information). Using the peak responsivity, the detectivity and noise equivalent power (NEP) of the device at room temperature are ≈4.5×10^11^ Jones and 8×10^−11^ W Hz^−1/2^, respectively. Although the performance of the DPD is close to that of low‐temperature (77 K) operating commercial InAs detectors (6×10^11^ Jones and 1.5×10^−13^ W Hz^−1/2^ at 77 K),^[^ ^46^ ^]^ our differential photodetector shows excellent performance without cryogenic cooling. Besides, the narrow‐bandgap of PbSe allows it to operate at longer wavelengths than InAs detectors (up to 3.1 µm).^[^ ^46^ ^]^ In **Table** **1**, we summarize the overall performance of traditional MISM photonic differentiator devices and heterojunction devices with transient responses. In addition to the conventional figures of merit for photodetectors, we introduce a new parameter *I_stable_/I_peak_* to better assess the signal differentiation function of DPDs. This parameter is the ratio of steady‐state photocurrent to peak photocurrent, where a smaller ratio indicates better differentiation capability.

**Table 1:** Figures of Merit of DPDs With Transient Response.

Structure	Wavelength [nm]	*τ_rise_/τ_fall_* [µs]	Detectivity [Jones]	*I_stable_/I_peak_*	Tunable Response	Refs.
ITO|PC|ZnPc|C60|PC|Au	639±10	−	−	≈0	NO	[47]
ITO|BDN:CB|PVDF|Al	1064	−	1.6 × 10^11^	≈0	NO	[30]
ITO|PC|ET‐PRBT|Ag	532	86/86	−	≈0	NO	[48]
ITO|VDF|nPc:C60|Al	532	−/≈100	‐	≈0	NO	[44]
n‐Si|n‐ZnO	532	110/110	4.0 × 10^11^	0.25 ^a)^	NO	[26]
Si/ZnO NWs	1064	15/21	8.78×10^11^	0.14 ^a)^	NO	[27]
MoS_2_/(BA)2PbBr4/BP	650	40/6000	−	≈0	NO	[14]
**This Work**	1550	0.013/0.489	4.5×10^11^	≈0	YES	

^a)^ Calculated from I‐t curves.

Compared with the listed DPDs with similar output waveforms, our detector has the capability to output transient response and normal continuous response by changing the bias voltage. Our DPDs can operate at longer wavelengths (see Figures S14 and S15, Supporting Information). The broadband response enables it to work in the ≈1 MHz frequency, making it promising for applications in optical communication as an optical receiver. Additionally, it can be used as an optical differentiator to shorten pulse duration and generate synchronized reset pulses for triggers or counters. Due to its low material cost and relatively simple fabrication process, it may also have potential applications in artificial retinas and event‐based visual sensing.^[^ ^49^, ^50^ ^]^ Besides, we further characterized the stability performance of the photocurrent response of our device with more than 3×10^5^ cycles of light pulses as shown in Figure S16 (Supporting Information). The device remained fully functional after being stored in the ambient environment for ten months, and there was no visible degradation after 3×10^5^ cycles of the light pulse test, demonstrating the remarkable stability of our device.

**Figure 5:**
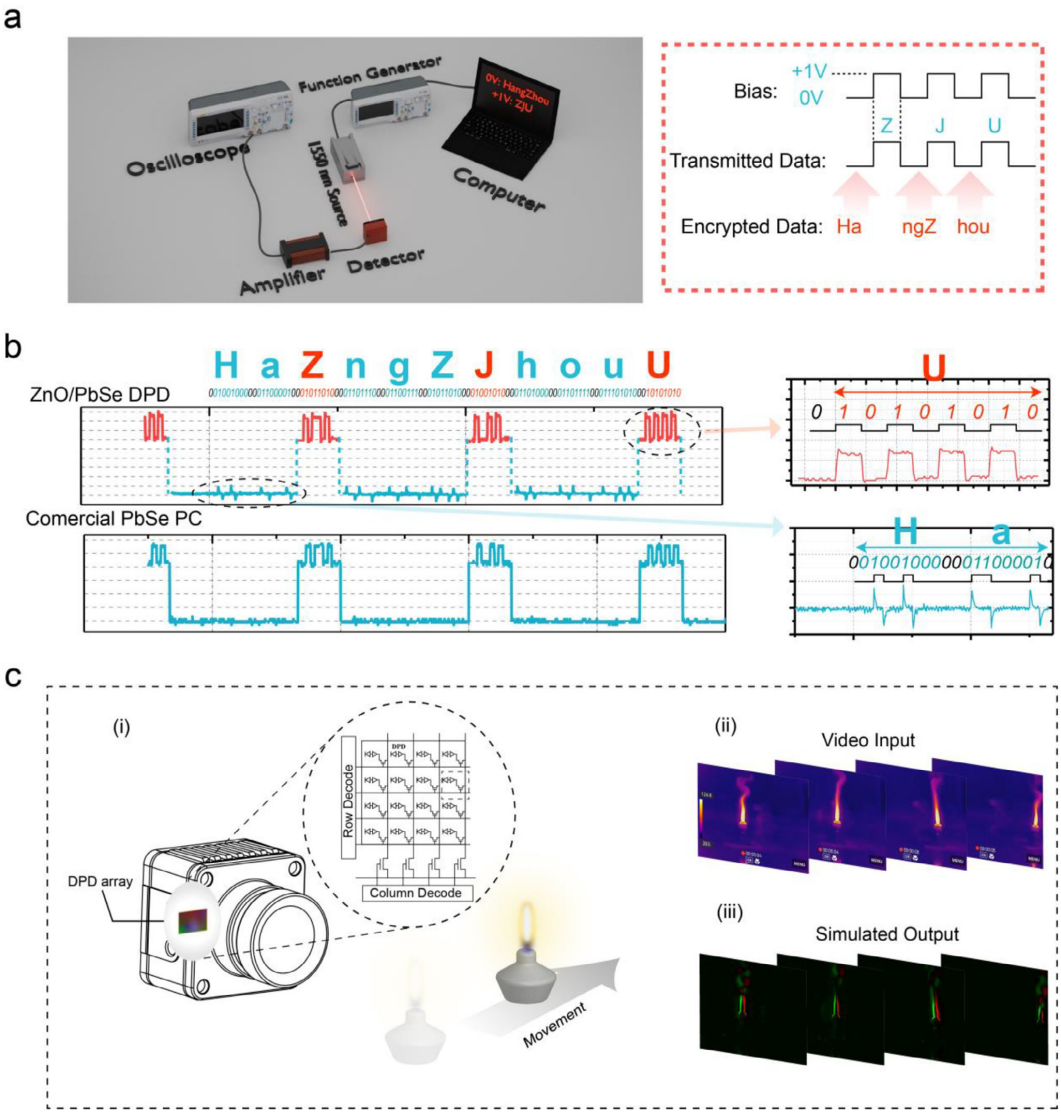
a) Schematic of optical communication. b) Comparison of the output signals between ZnO/PbSe DPD and commercial PbSe PC. c) (i) Scenarios of event cameras based on DPD; (ii) IR camera images of a moving flame; (iii) Simulated output of a 2D DPD array of the differential images extracted from the IR camera.

Since the detector can respond to the changes in optical power density at a high speed and high responsivity, it is expected to be used as an event‐based camera or applied to optical communication. **Figure** **5**a is a schematic of optical communication. We input “ZJU” in binary ASCII code into the driver of a 1550 nm laser. For example, the binary code for the letter “Z” is “01011010”. By applying a pulse bias, the device operates in the “normal mode” to transmit data, while encrypted data “HangZhou” is embedded in the adjacent pulses in ASCII code form, as shown in Figure 5a. Figure 5b presents the device's output results. For comparison, the output from a commercial PbSe photoconductive (PC) detector under the same test conditions is provided. The PbSe PC detector cannot detect the encrypted data between two pulses. By contrast, the ZnO/PbSe DPD can operate in the ‘differential mode’, where the positive and negative signals from the detector correspond to the rising and falling edges of the optical pulses, respectively. Considering that reading such signals requires a special protocol, the detector can be applied to the transmission of encrypted signals. Besides, we also demonstrate real‐time monitoring of the high speed of a fan by the detector (See Figure S17, Supporting Information). Finally, we demonstrate the application scenarios in event cameras, and the imaging schematic for the moving flame is shown in part (i) of Figure 5c. We took a video of the moving flame using an IR camera (at a frame rate of 25 Hz), and then used the video data as the input for the simulation, shown in part (ii) of Figure 5c. Based on the model from reference,^[^ ^51^ ^]^ our simulated response output of the 2D DPD array is shown in part (iii) of Figure 5c. The simulation details and principles of event cameras with tunable operation modes are provided in the supplementary text and Figure S18 (Supporting Information). The above results demonstrate that the readout circuit in each frame of event cameras only needs to process pixels where the light intensity has changed, rather than scanning all pixels in every frame as in a conventional camera. As a result, the event cameras could capture fast‐moving objects and omit the saving of redundant data of unchanged‐intensity frame pixels.

## 3 Conclusion

We invented a novel heterojunction made of wide‐bandgap ZnO with n‐type and narrow‐bandgap PbSe with p‐type and successfully developed a ZnO/PbSe DPD. Through *C‐V* characterization, a large conduction band offset (≈0.47 eV) at the ZnO/PbSe interface was revealed. The unique band alignment and the accumulation and recombination of photo‐generated carriers played key roles in the DPD operation. By adjusting the band alignment at the ZnO/PbSe interface through changing the bias voltages, the device could be freely switched between “differential” and “normal” modes. This tunability allowed the device to function both as a signal differentiator and a normal response photodetector. Due to the narrow‐bandgap of PbSe, the device operates at longer wavelengths than reported to date. Furthermore, the device exhibited a broad bandwidth of 1 MHz, high detectivity of 4.5×10^11^ Jones, and low NEP of 8×10^−11^ W Hz^−1/2^ under zero bias at room temperature. Applications of encrypted communication and real‐time monitoring of a fan speed were also demonstrated by the device. Scenarios of event cameras based on DPD were also demonstrated. This study opens new possibilities and demonstrates significant potential for a wide range of applications in multifunctional integrated optical systems.

## 4 Experimental Section

### Film Deposition

ZnO Thin Film Preparation: The ZnO was grown using a magnetron sputtering method. The target used for sputtering was of high purity, and the growth occurred in a high‐vacuum magnetron sputtering chamber. After degassing by heating to 100 °C for 15 min, the substrate was cooled to room temperature. The ZnO target was pre‐sputtered for 5 min. During sputtering, the argon pressure was maintained at 1.6 Pa with a power of 150 W. The ZnO growth was performed with a thickness range of 100–400 nm. PbSe Thin Film Preparation: The PbSe thin film was prepared using a chemical bath deposition method. First, 3.2 mL of lead acetate trihydrate solution and 4.7 mL of TSC (thiourea) solution were added to a centrifuge tube and stirred thoroughly until the white flocculent precipitate disappeared. This lead acetate and TSC mixture was then poured into a reaction vessel, followed by the addition of 30.2 mL of deionized water, and stirred magnetically for 5 min. Next, 1.5 mL of potassium hydroxide solution was added and stirred for another 5 min. Subsequently, 0.06 g of KI was dissolved in 5 mL of deionized water, and this solution was poured into the reaction vessel and stirred for 5 min. Finally, 5.4 mL of sodium selenosulfate solution was added and stirred for 5 min. The reaction vessel was then covered with a glass lid and heated in a 65 °C water bath for 3 h. After the reaction, the sample was washed with deionized water and dried with a nitrogen gun.

### Device Fabrication

In the deposited ZnO/PbSe heterojunction photodetectors, the ZnO layer thicknesses are 100, 200, and 400 nm, respectively, while the PbSe layer is 1 µm thick. Concentric circular mesas with diameters ranging from 50 to 400 µm were patterned using photolithography and dry etching. To prevent short circuits between the top electrode and the sidewalls of the underlying PbSe, the sidewall areas were filled with epoxy resin. Considering that Ti/Au can form Ohmic contacts with both ZnO and PbSe, Ti (5 nm)/Au (100 nm) contacts were deposited as electrodes using electron beam evaporation.

### Electrical and Optoelectric Properties Measurements

The current‐voltage (*I‐V*) characteristics of the device were measured using a Keysight B1500A semiconductor analyzer. The capacitance‐voltage (*C‐V*) characteristics were measured with an impedance analyzer (HIOKI 3532–50 LCR) and a source meter (Keithley 2612A). Transient photocurrent measurements were performed using a wavelength‐tunable femtosecond (fs) Yb: KGW laser (2000 nm, 220 fs Gaussian fit, 100 kHz). For the measurements of optoelectronic characteristics at a wavelength of 1064 nm, a diode‐pumped solid‐state (DPSS) laser at 1064 nm was used. The laser was controlled by a laser driver (VD‐IIIA DPSS Laser Driver) with a maximum frequency of 20 kHz or switched by an optical chopper (SR540, SRS) with a maximum frequency of 400 Hz. For frequency‐dependent measurements at 1550 nm, a 1550 nm DFB laser diode (TM‐DB3CI431, Innoconn Sensing Technologies) with an output power density of ≈2.4 W cm^−2^ was used. This laser diode was modulated by a function generator (AFG1022, Tektronix) with a maximum frequency of 10 MHz. The laser was focused onto the detector using a calcium fluoride convex lens, and the signal was amplified by a high‐speed current amplifier (DHPCA‐100, FEMTO) and displayed on an oscilloscope (TBS1052B, Tektronix).

## Conflict of Interest

The authors declare no conflict of interest.

## Supporting information



Supporting Information

## Data Availability

The data that support the findings of this study are available from the corresponding author upon reasonable request.
