# Supplementary material for: Wide‐/Narrow‐Bandgap Heterojunction for High Performance Differential Photodetector with Tunable Response
Source: Adv Sci (Weinh). 2025 Jul 29;12(40):e04872. doi: 10.1002/advs.202504872 (PMC12561176; doi:10.1002/advs.202504872)
Supplement: Supplementary file 1 — Supporting Information [file ADVS-12-e04872-s001.docx]

**Supplementary information**

**Wide-/Narrow-Bandgap Heterojunction for High Performance Differential Photodetector with Tunable Response**

*Ziyang Ren, Haimin Zhu, Weien Lai*, Yihui Zhai, Mengjuan Liu, Yu Zhu, Hanlun Xu, Nasir Ali, Ning Dai, Jiaqi Zhu*, Sihan Zhao*, Huizhen Wu**

**Supplementary Text**

Calculation details of the band offset :


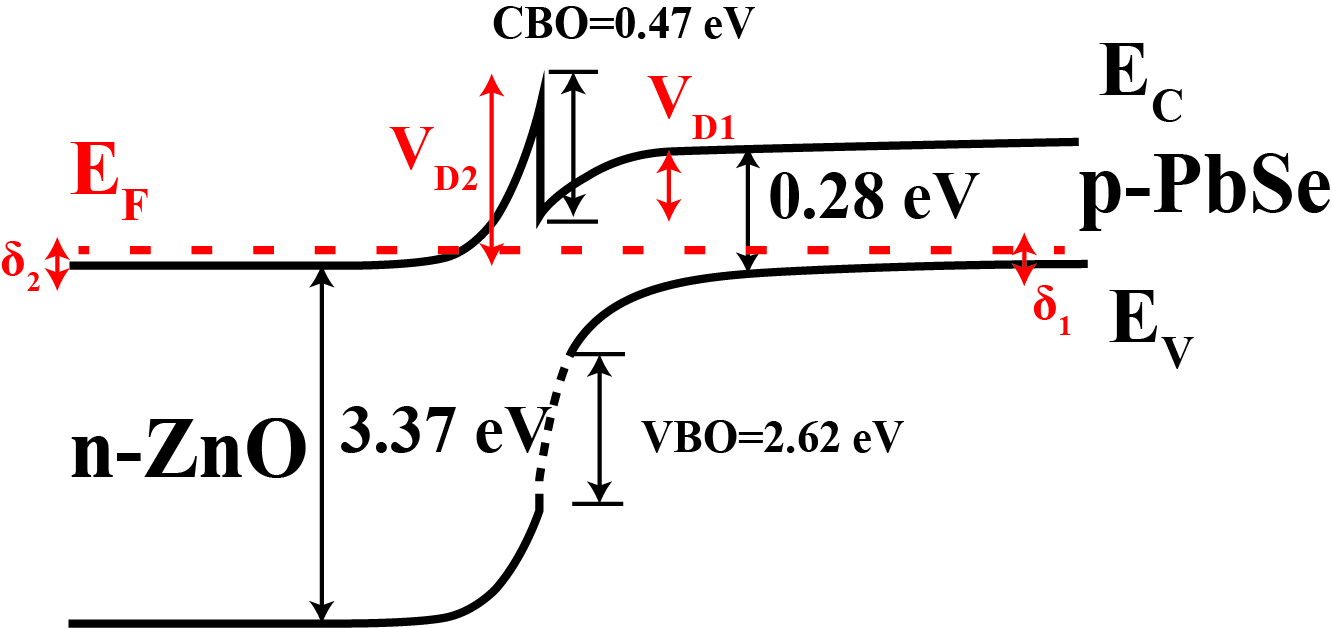


**Figure S1:** Energy band alignment of the n-ZnO/p-PbSe heterojunction.

Based on the Anderson model^[1]^:

$$\begin{aligned} \frac{1}{C^{2}}=2\frac{\epsilon_{1}N_{A}+\epsilon_{2}N_{D}}{q\epsilon_{1}\epsilon_{2}N_{A}N_{D}}\left( V_{D}-V \right)\#\left( 1 \right) \end{aligned}$$

In the equation, ε_1_ and ε_2_ are the dielectric constants of the PbSe and ZnO, respectively. N_A_ and N_D_ represent the acceptor and donor concentrations of the PbSe and ZnO, respectively. V_D_ is the barrier height, which equals 0.76 ± 0.053 eV in Figure 1 (c).

From the band alignment, it can be seen that^[2]^:

$$\begin{aligned} \Delta E_{C}=qV_{D2}+\delta_{2}-\left( E_{g1}-qV_{D1}-\delta_{1} \right)=qV_{D}+\delta_{2}+\delta_{1}-E_{g1}\#\left( 2 \right) \end{aligned}$$

Here, $qV_{D2}$ and $qV_{D1}$ represent the potential barrier distribution on the ZnO and PbSe sides, respectively. while $\delta_{1}$ and $\delta_{2}$ present the positions of the Fermi level relative to the valence band of PbSe and the conduction band of ZnO. $E_{g1}$ is the energy gap on the PbSe side. Assuming that all donors and acceptors on both sides of the interface are fully ionized, we can use the Fermi distribution to analyze the system. From this, we can derive the relationship between the electron concentration on the ZnO side and the Fermi level^[3]^.

$$\begin{aligned} n_{0}=2\left( \frac{m_{n}k_{0}T}{2\pi\hbar^{2}} \right)^{\frac{3}{2}}*\frac{2}{\sqrt{\pi}}F{}_{\frac{1}{2}}\left( \frac{-\delta_{2}}{k_{0}T} \right)\#\left( 3 \right) \end{aligned}$$

The equation includes $m_{n}$, the effective mass of electrons on the ZnO side, $k_{0}$, the Boltzmann constant, $\hbar$, the reduced Planck constant, and $F_{\frac{1}{2}}(x)$, the Fermi-Dirac integral:

$$\begin{aligned} F_{\frac{1}{2}}\left( x \right)=\int_{0}^{\infty} \frac{t^{\frac{1}{2}}}{1+e^{t-x}}dt\#\left( 4 \right) \end{aligned}$$

Similarly, the relationship between the hole concentration in PbSe and the Fermi level can be derived:

$$\begin{aligned} p_{0}=2\left( \frac{m_{p}k_{0}T}{2\pi\hbar^{2}} \right)^{\frac{3}{2}}*\frac{2}{\sqrt{\pi}}F{}_{\frac{1}{2}}\left( \frac{-\delta_{1}}{k_{0}T} \right)\#\left( 5 \right) \end{aligned}$$

The equation includes $m_{p}$, the effective mass of holes on the PbSe side. By solving the above two equations using numerical methods, $\delta_{1}$ and $\delta_{2}$ are found to be -0.145 eV and 0.08 eV, respectively. Substituting these values into the first equation, the conduction band offset $\Delta E_{C}$ is calculated to be 0.47 eV, and then according to the formula:

$$\begin{aligned} \Delta E_{V}=\left( E_{g2}-E_{g1} \right)-\Delta E_{C}\#\left( 6 \right) \end{aligned}$$

The valence band offset is calculated to be 2.62 eV, where $E_{g1}$ and $E_{g2}$ represent the band gaps of PbSe and ZnO, respectively.

The general formula related to device performance:

The following equation is provided in the main text:

$$\begin{aligned} I{}_{out}+\left( R_{m}^{'}+R_{0} \right)C_{s}\frac{dI_{out}}{dt}={C_{s}R}_{m}^{'}\frac{dI_{ph}}{dt}\#\left( 7 \right) \end{aligned}$$

Considering that$I_{ph}$ is proportional to the irradiated light power $\Phi$ and the quantum efficiency $\eta$, approximating $\frac{dI_{ph}}{dt}$ as an impulse function and solving the equation can yield:

$$\begin{aligned} I_{out}=\left\{ \begin{aligned} k\eta\Phi\frac{R_{m}^{'}}{R_{0}+R_{m}'}e^{-\frac{t}{{(R}_{0}+R_{m}')C_{s}}}, upon light on \\ -k\eta\Phi\frac{R_{m}^{'}}{R_{0}+R_{m}'}e^{-\frac{t}{{(R}_{0}+R_{m}')C_{s}}}, upon light off \end{aligned} \right.\#\left( 8 \right) \end{aligned}$$

In this equation, k is a proportionality factor, and the value of $R_{m}^{'}$ is generally very small and can be neglected. Considering that C­­_S_ and $\Phi$ are related to the area of the device, for simplicity, we assume that $\Phi$ is proportional to the device area, and Cs has a linear relationship with the area. From Equation S9 we can get:

$\begin{aligned} I_{out}\left( \max\right)=k\eta\Phi\frac{R_{m}^{'}}{R_{0}+R_{m}'}\propto A\#\left( 9 \right) \end{aligned}$

$\begin{aligned} BW=\frac{1}{2\pi\left( R_{m}^{'}+R_{0} \right)C_{s}}\propto\frac{1}{A+C_{0}}\#\left( 10 \right) \end{aligned}$

A is the photosensitive area of the device. C_0_ is a constant, meaning the part that does not change with the area.

For a photodetector, two Figures of merit are its detectivity and noise equivalent power (NEP). The noise equivalent power is defined as the optical input power required for the output signal to reach the same level as its noise. It can be calculated using the following equation:

$$\begin{aligned} NEP=\frac{\left( A\Delta f \right)^{\frac{1}{2}}}{D^{*}}\#\left( 11 \right) \end{aligned}$$

A is the effective area of the photodetector, $\Delta f$ is the electronics bandwidth, and $D^{*}$ is the detectivity, with units of Jones. If we consider the case where shot noise dominates, $D^{*}$ can be expressed by the following equation:

$$\begin{aligned} D^{*}=\frac{R_{i}}{\left( 2qI_{D} \right)^{\frac{1}{2}}}\#\left( 12 \right) \end{aligned}$$

Here, q is the elementary charge, and I_D_ is the device's dark current. R_i_ is the photoresponsivity, which is extracted using the formula:

$$\begin{aligned} R_{i}=\frac{I_{ph}}{P_{light}S}\#\left( 13 \right) \end{aligned}$$

Where I_ph_ represents the photocurrent of the photodetector, S refers to the area of the detector, and P_light_ represents the light density.

Quantitative description of the simulation:

From equation (7) and model form ^[4]^, considering that the response time of the device is much smaller than the time interval between frames (25 ms), the first term on the left side of Equation (7) can be removed, simplifying it to:

$$\begin{aligned} I_{out}={C_{s}R}_{m}^{'}\frac{dI_{ph}}{dt}\#\left( 14 \right) \end{aligned}$$

Considering the relationship between I_ph_ and optical power P:

$$\begin{aligned} I{}_{ph}=I_{0}P^{\gamma}\#\left( 15 \right) \end{aligned}$$

For simplicity, we take γ as 1, so we have:

$$\begin{aligned} I_{out}=K_{0}\frac{dP}{dt}\#\left( 16 \right) \end{aligned}$$

Where K_0_ is a proportionality factor, so that we can express the above equation in differential form:

$$dP\to\Delta P= P_{i}-P_{i-1}$$

$$\begin{aligned} I_{out,i}=K_{0}\times\frac{\Delta P}{\Delta t}\#\left( 17 \right) \end{aligned}$$

**Supplementary Figures**


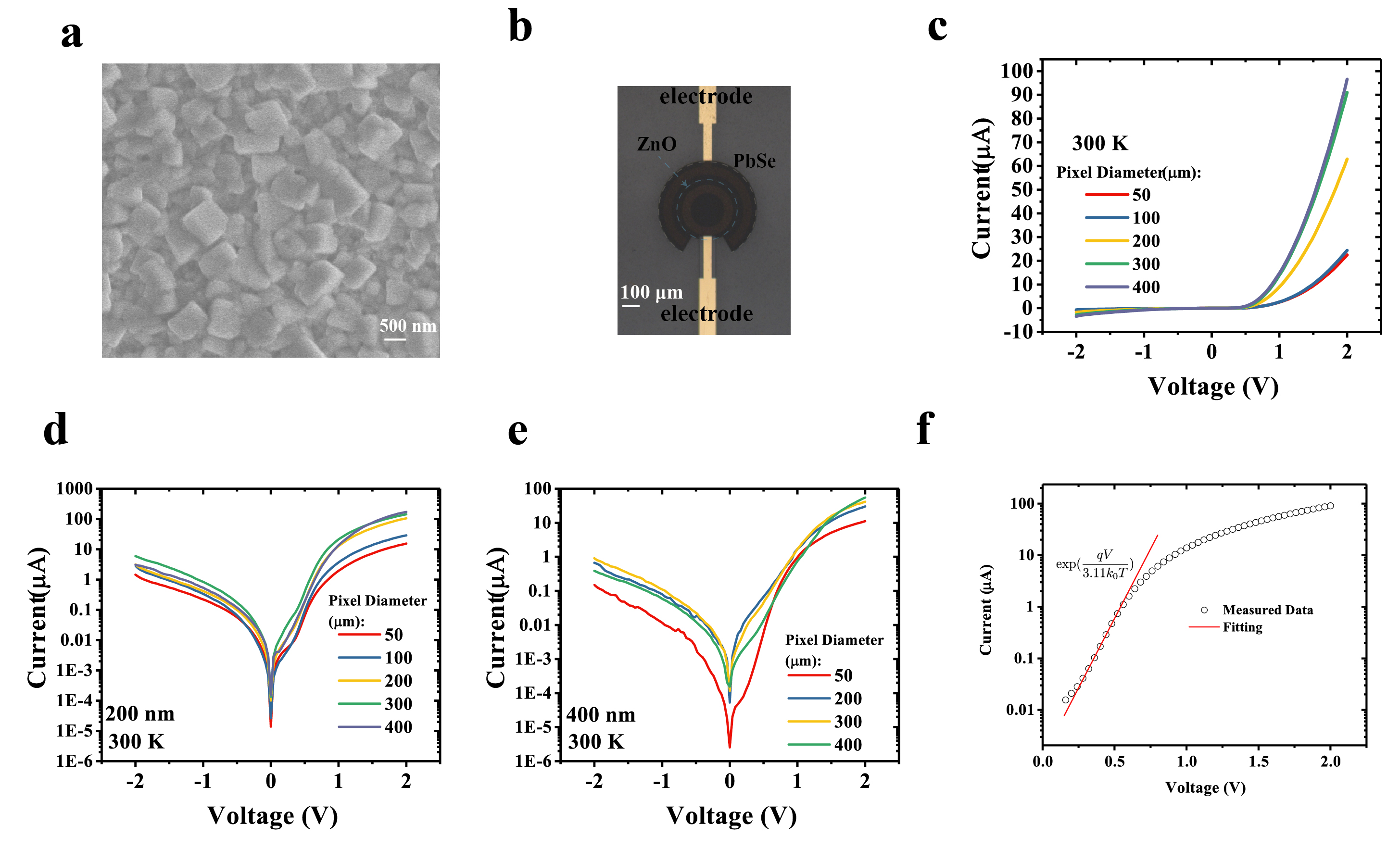


**Figure S2:** Image and I-V curves of ZnO/PbSe detector. (a) SEM image of PbSe. (b) Optical microscope image of the ZnO/PbSe heterojunction. (c) I-V characteristic curves of ZnO(100nm)/PbSe(1 μm) with different sizes (in geometric coordinates). (d) I-V characteristic curves of ZnO(200nm)/PbSe(1 μm) with different sizes. (e) I-V characteristic curves of ZnO(400nm)/PbSe(1 μm) with different sizes. (f) Fitting of the forward bias I-V curves of the ZnO/PbSe heterojunction.

Though many factors could affect the ideality factor n in a traditional diode according to dark current formula:$I\propto\exp\left( {qV}/{nk_{0}T} \right)-1$. In our device, interface defects are a primary factor. The interface defects impact device performance by either trapping charge carriers or forming transport channels at the interface. If the defect density at the interface is too high, it not only reduces the separation efficiency of photogenerated electron-hole pairs but also significantly increases the device's dark current and noise. Therefore, improving the ideality factor of a photodiode not only significantly enhances device performance but also aids in analyzing related experimental phenomena.


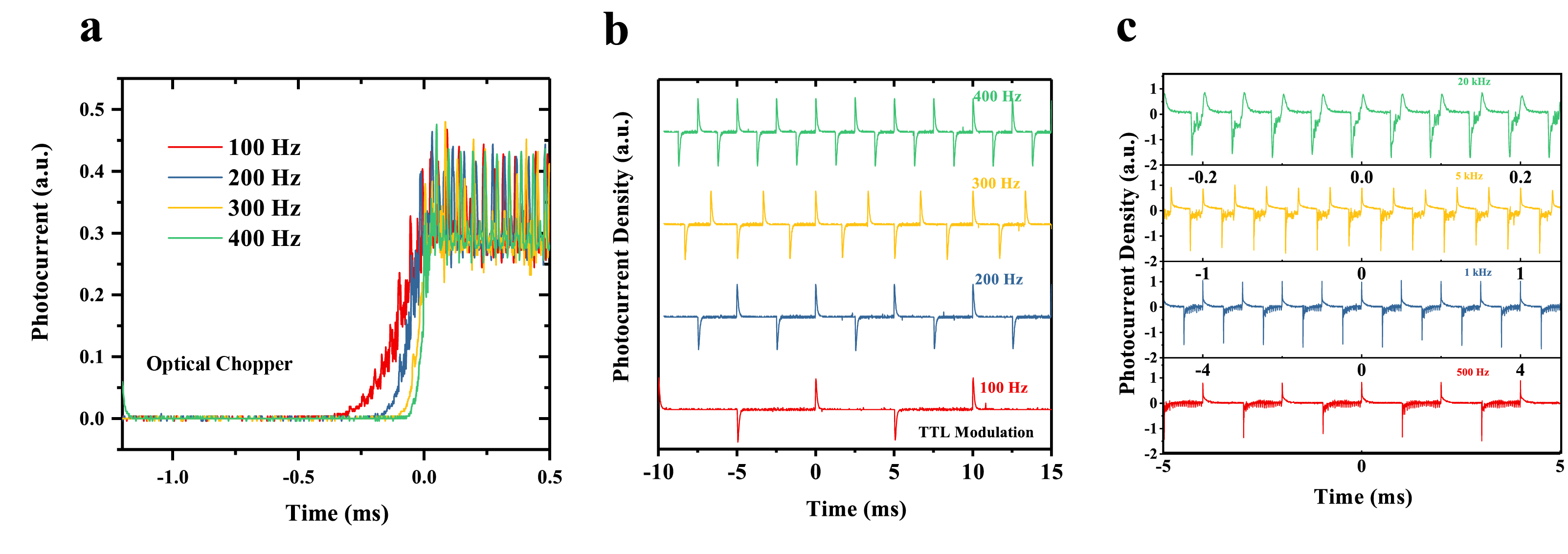


**Figure S3:** (a) The rising edge of the 1064 nm laser under optical chopper at a frequency of 100-400 Hz; (The detector used is an InGaAs pin photodiode, with a responsivity of 1 A/W, response time of 1 ns, amplifier gain of 10^3^ V/A, and bandwidth of 200 MHz). (b) I-t curve of the device under TTL modulation with a 1064 nm laser at a frequency of 100-400 Hz. (c) I-t curve of the device under TTL modulation with a 1064 nm laser at a frequency of 0.5-20 kHz;


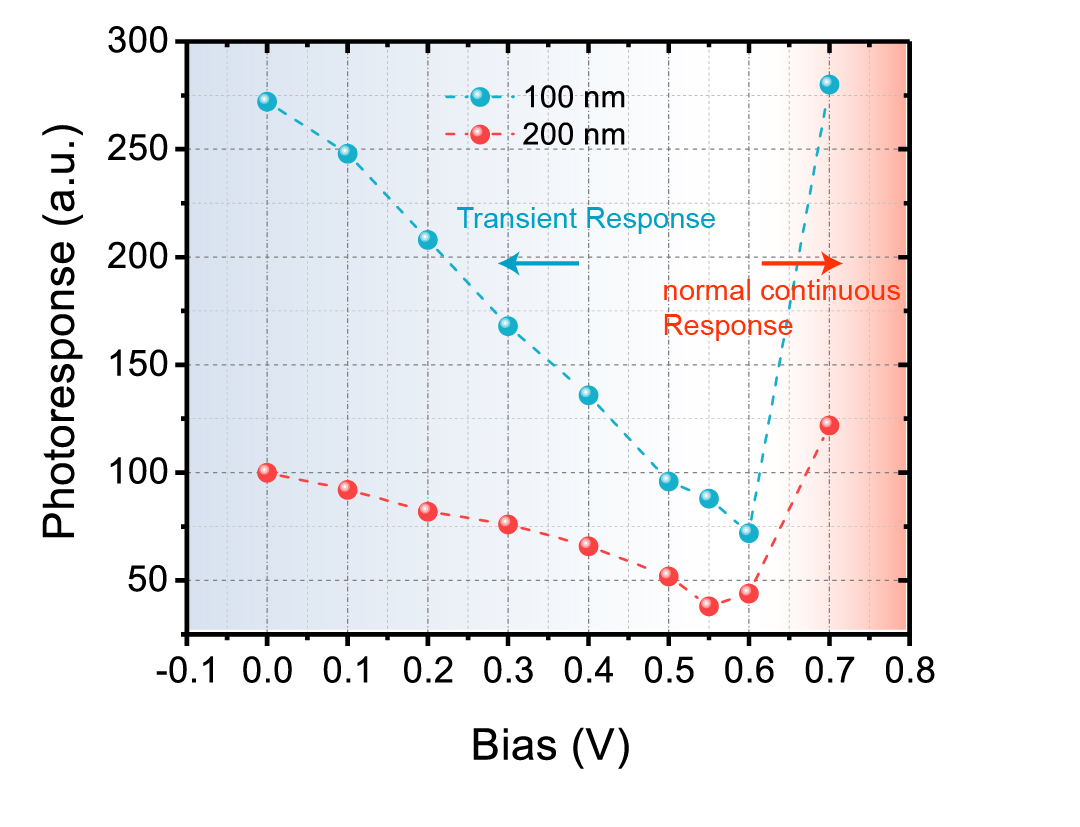


**Figure S4:** The photocurrent response output of the device under different positive biases.


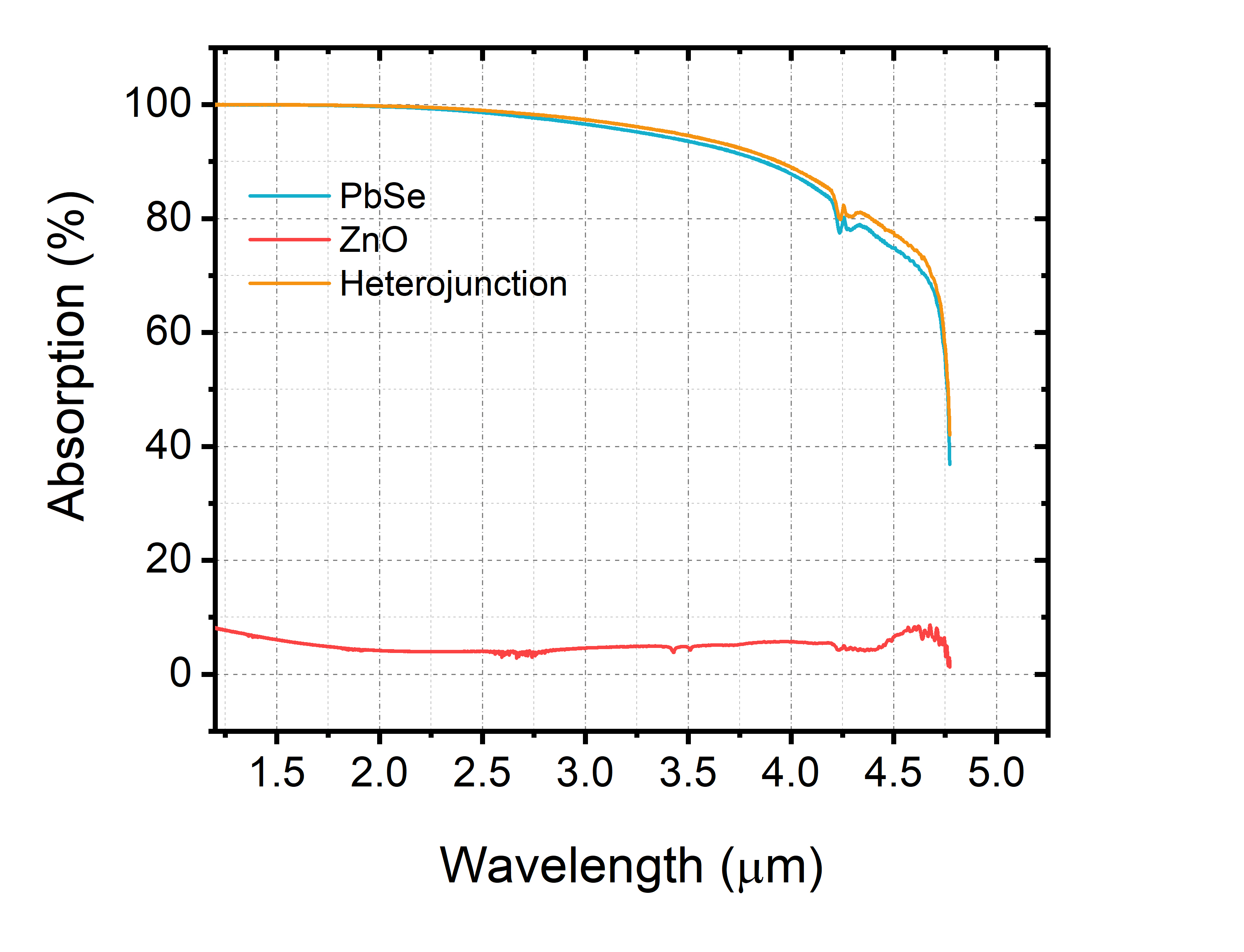


**Figure S5:** Absorption spectra of ZnO(100 nm), PbSe, and ZnO/PbSe heterojunctions in the infrared band.

**Figure S6:** Simulation results by TCAD. (a) The band alignment calculation results of ZnO/PbSe under different concentrations of interface negative charge (2-8 × 10^12^ cm^-2^). (b) The built-in electric field distribution map of the ZnO/PbSe heterojunction under different concentrations of interface negative charge.


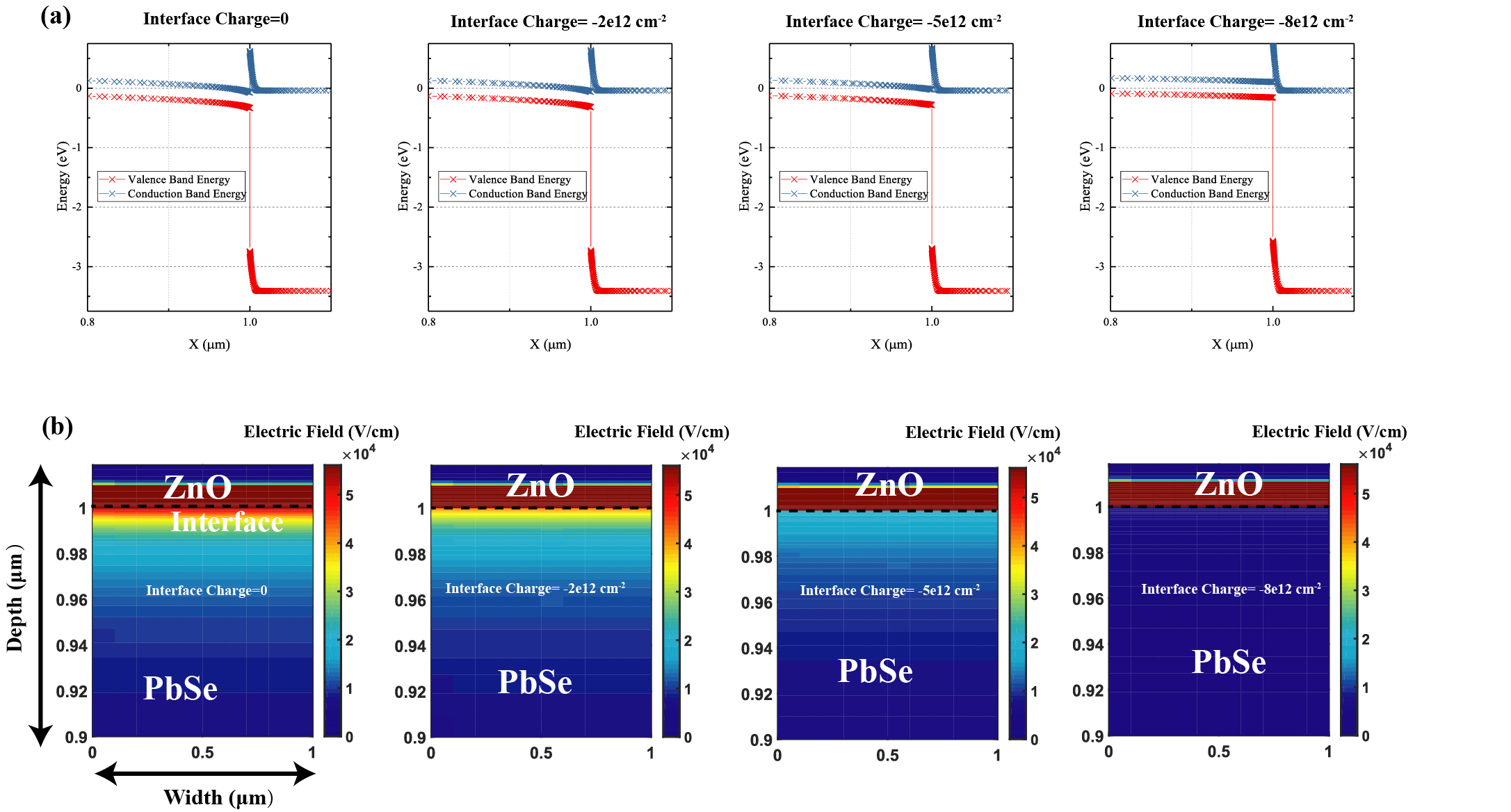

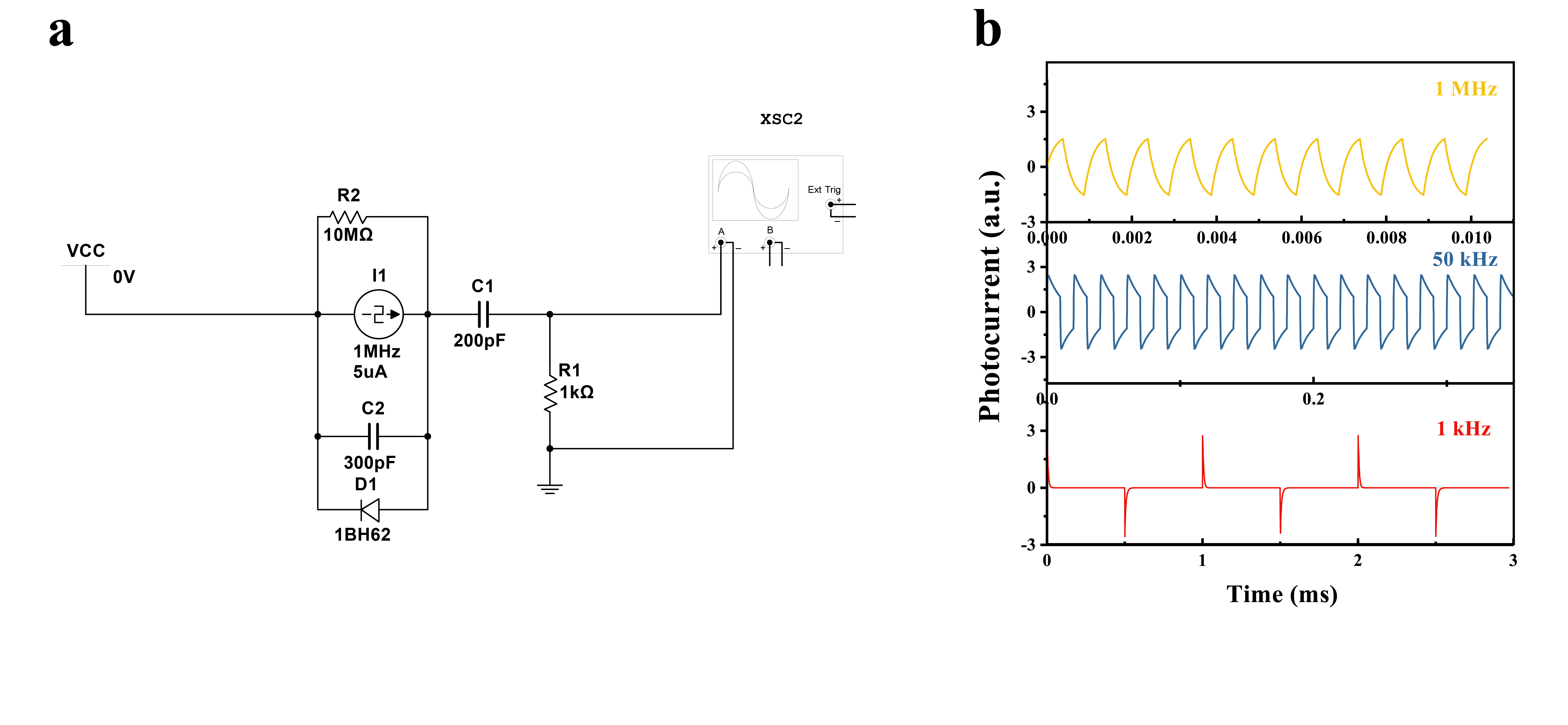


**Figure S7:** Multisim schematic and simulation results. (a) Circuit diagram: The left part represents the equivalent circuit of the pn junction. In this circuit, R2 is the leakage current resistor, I1 is the current source, C2 is the space charge region capacitance, and D1 is the diode. Additionally, C1 represents the equivalent capacitance, and R1 is the load resistor. The current flowing through the load resistor is recorded via an oscilloscope. The specific values of the equivalent circuit components are estimated from the I-V and C-V curves. (b) I-t curves of the current flowing through the load resistor at different frequencies.


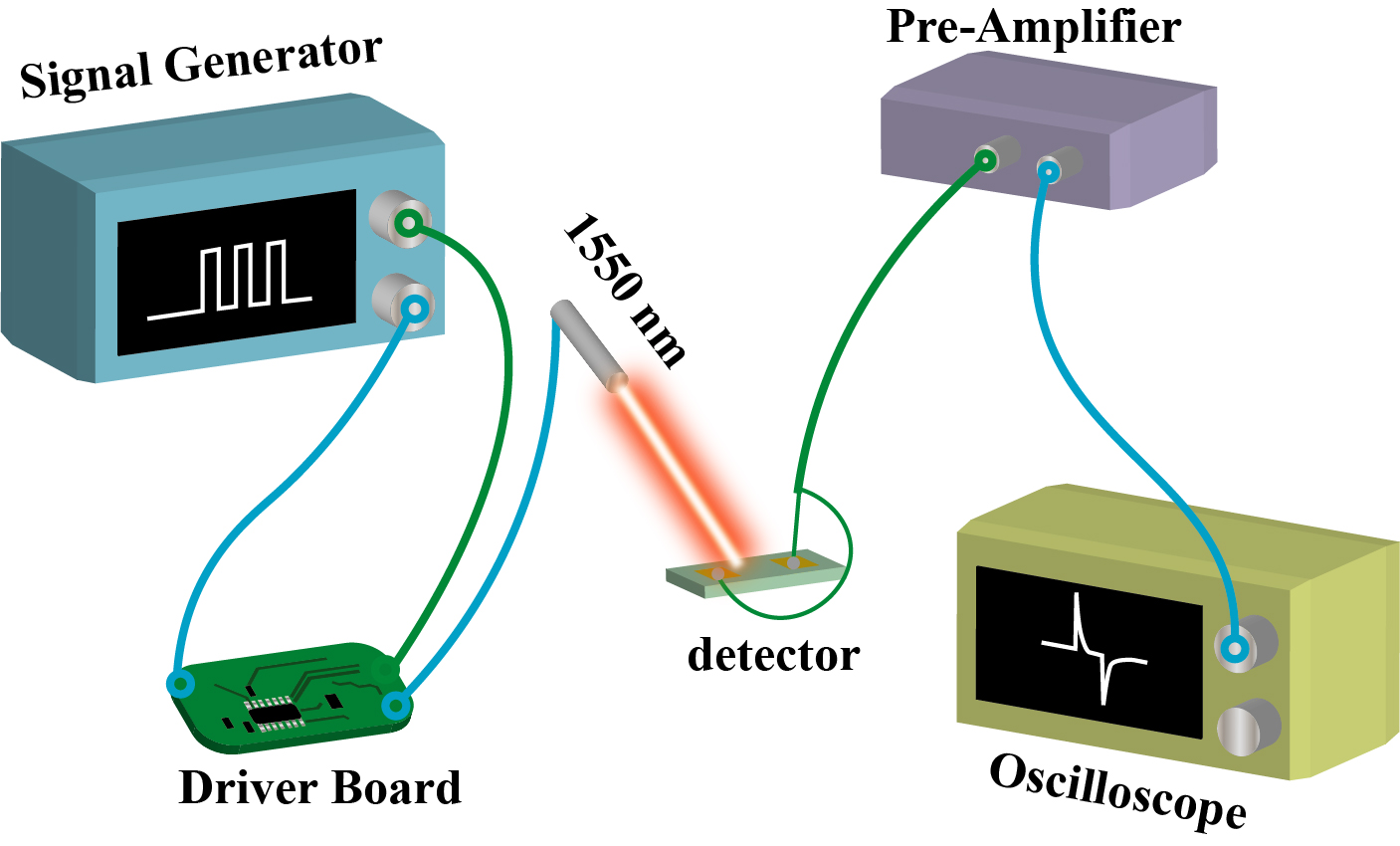


**Figure S8:** Schematic of the experiment set-up for bandwidth measurement.


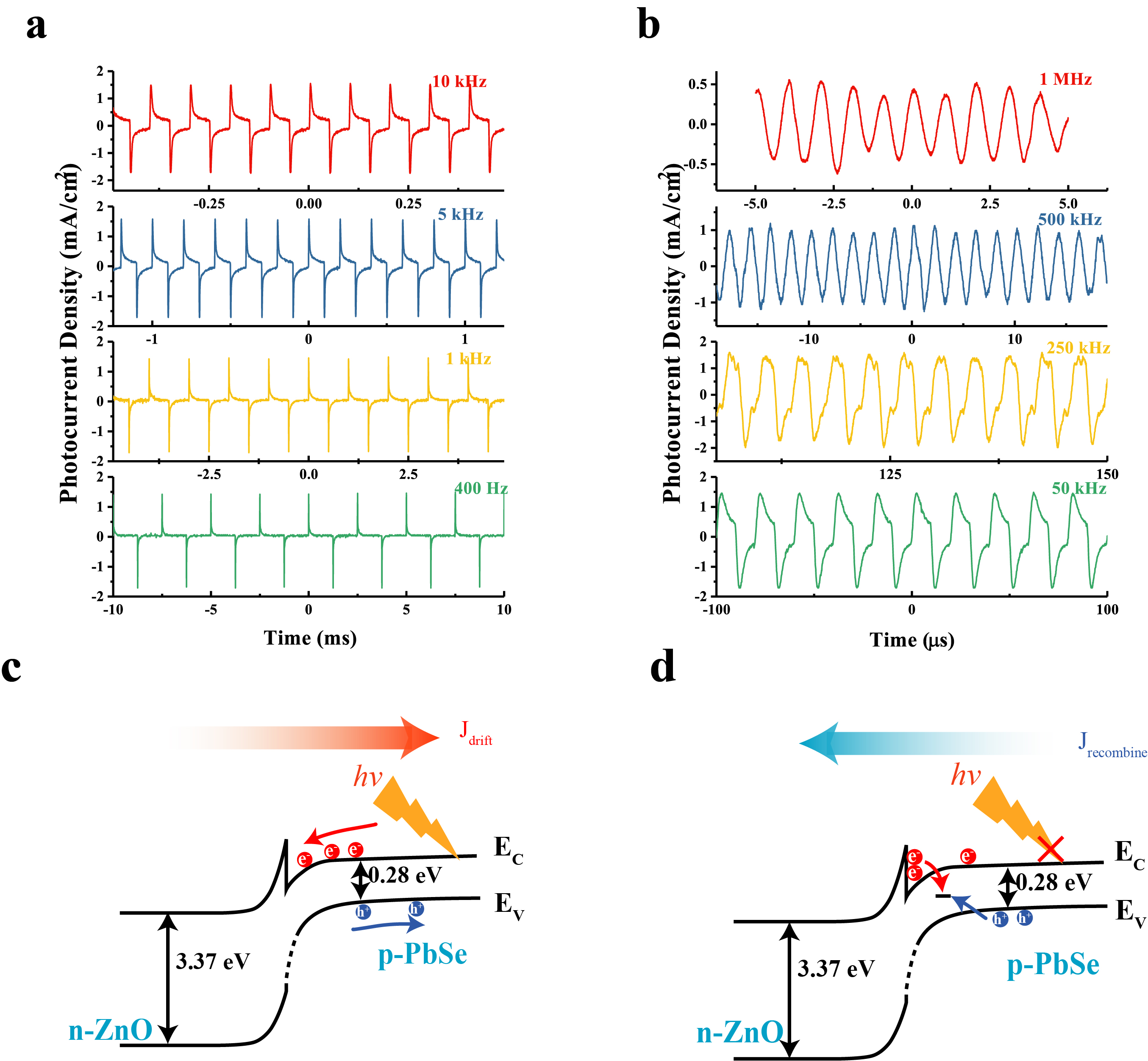


**Figure S9:** (a) I-t curve of the device under electrical modulation with a 1550 nm laser at a frequency of 400-10 kHz. (b) I-t curve of the device under electrical modulation with a 1550 nm laser at a frequency of 50 kHz-1 MHz. (c) Energy band diagram when illumination is turned on. (d) Energy band diagram when illumination is turned off.

To illustrate the waveform of the device under high illumination frequency, we have drawn the energy band diagrams as shown in (c) and (d). Since the illumination changes very quickly (>250 kHz), neither the drift electrons nor the recombination process has reached an equilibrium state. Therefore, a triangular waveform, as seen in Figure S9(b), appears under high illumination frequency.


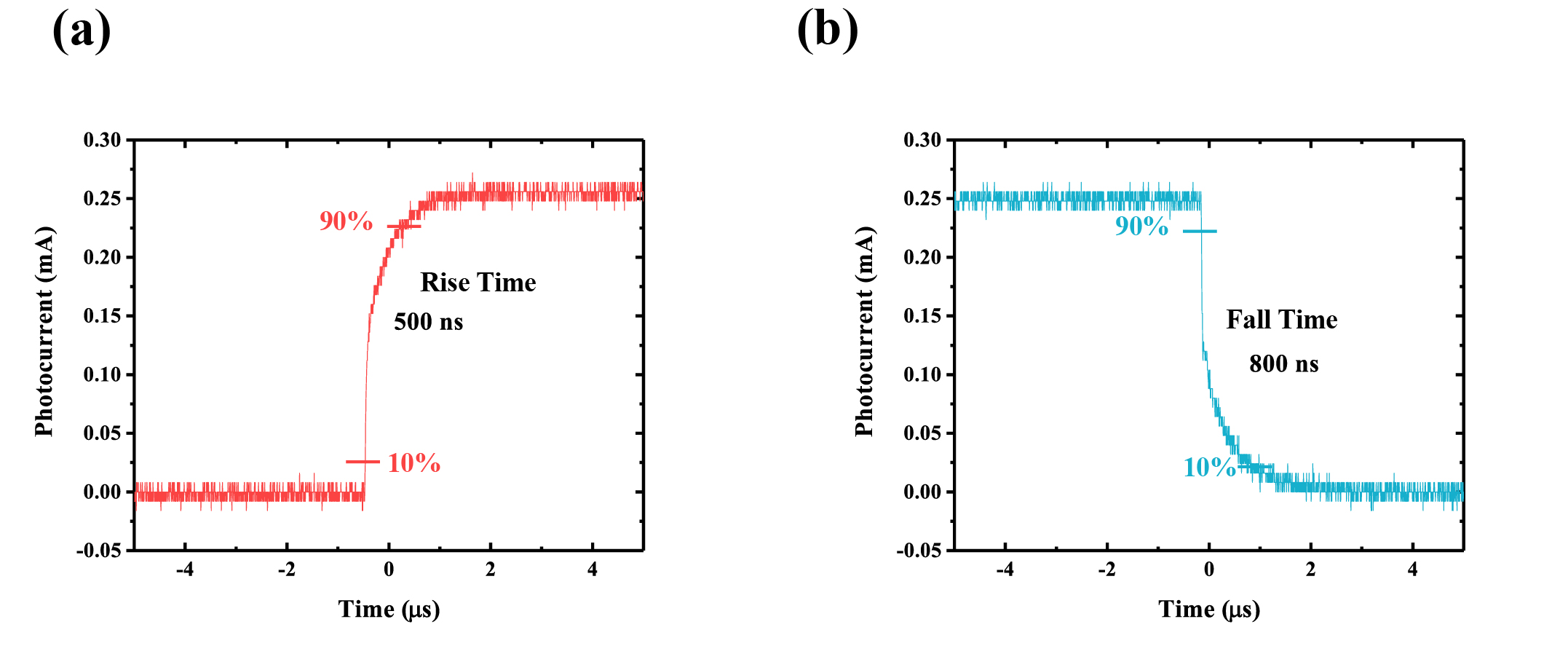


**Figure S10:** Rise/Fall time of 1550nm laser diode measured by InGaAs pin detector (response time:1ns) with an amplifier (Bandwidth: 80MHz).


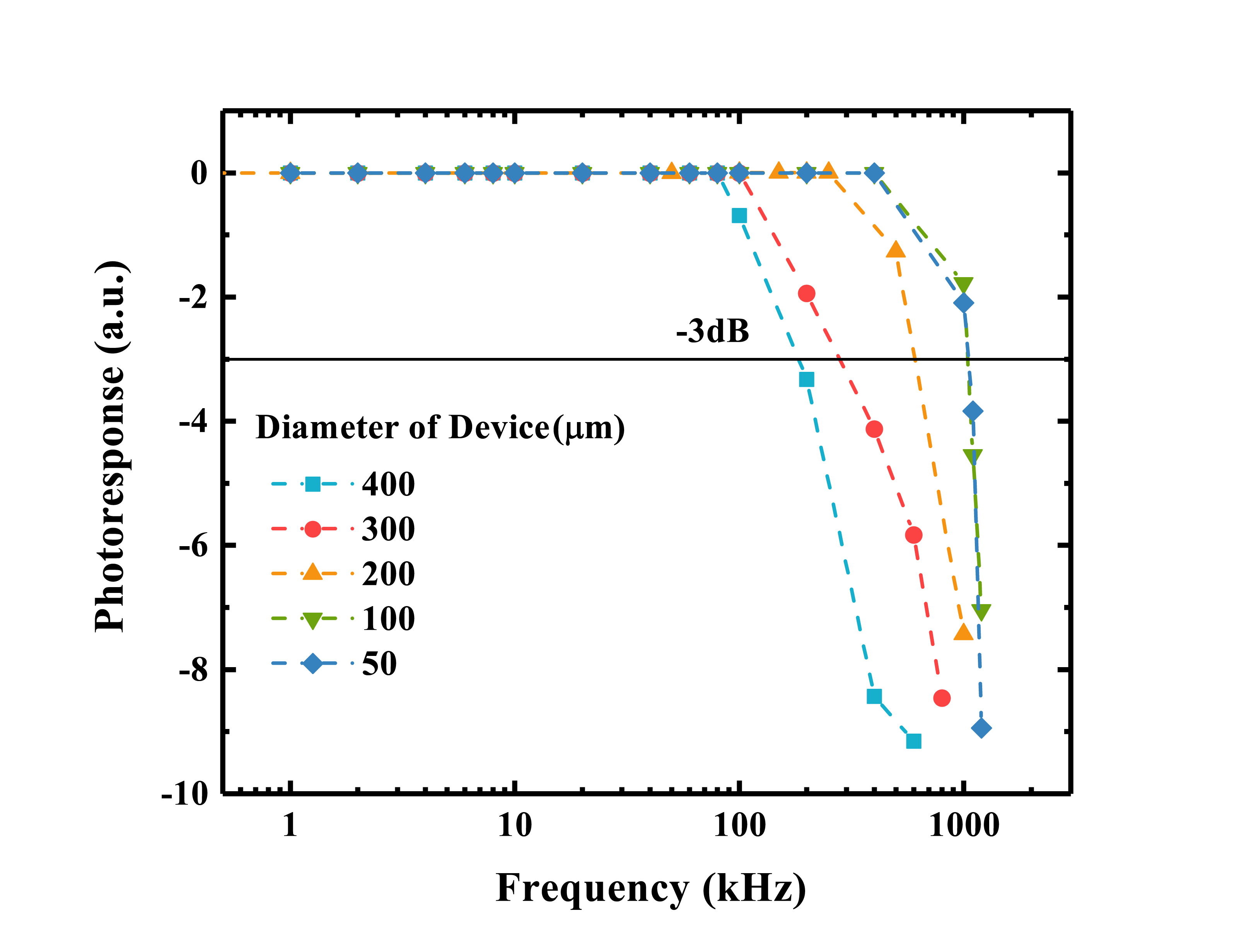


**Figure. S11:** Bode plot of the photoresponse of the detector with different device sizes.


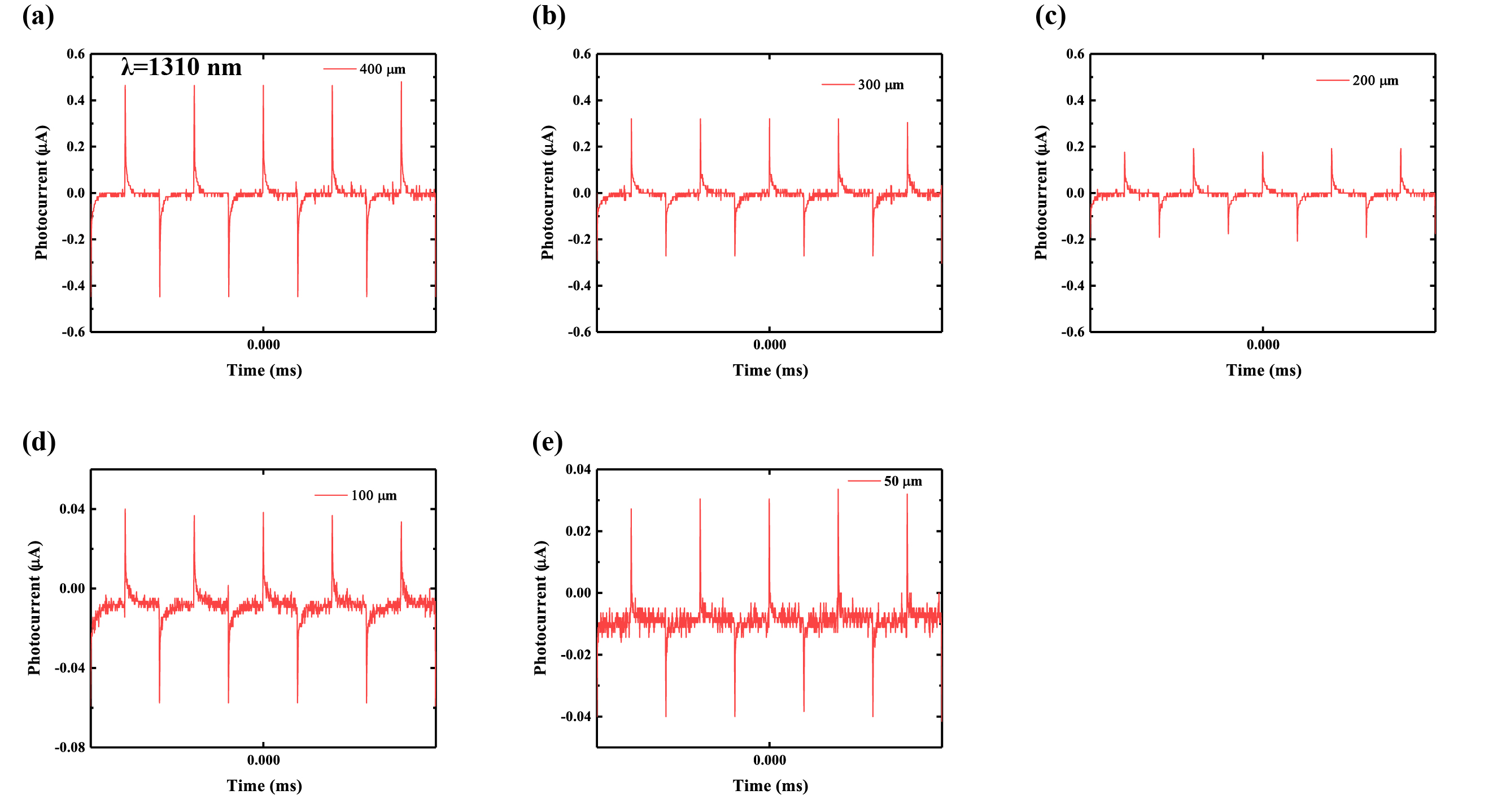


**Figure. S12:** Photocurrent of the ZnO/PbSe detectors with different photosensitive areas under the same irradiation density of 1310 nm light source.


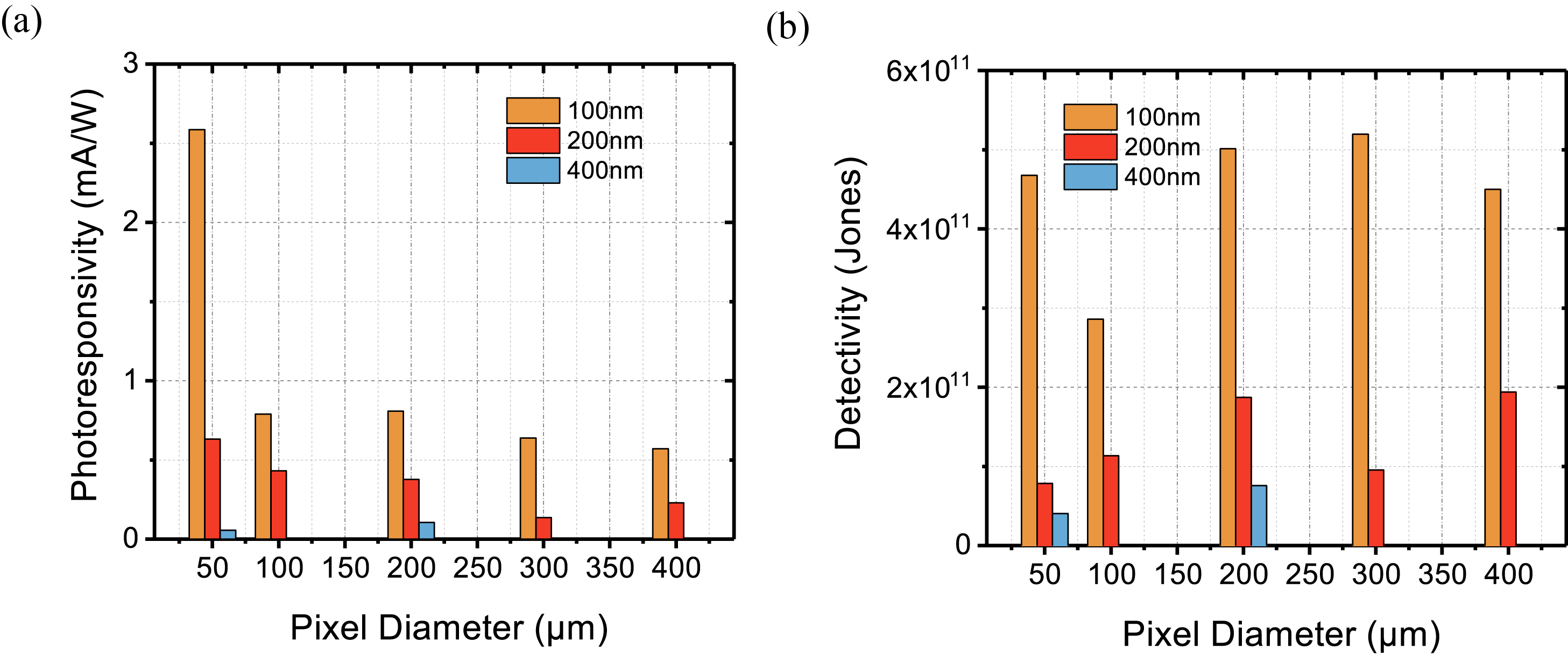


**Figure S13**: Performances of the devices at λ=1550 nm with different device sizes and different ZnO thicknesses.

**Table S1:** Summary of device responsivity and detectivity for varied sizes and ZnO thicknesses at λ=1550 nm

| **Thickness of ZnO**  **[nm]** | **Pixel Diameter**  **[μm]** | **Responsivity**  **[mA/W]** | **Detectivity**  **[Jones]** |
| --- | --- | --- | --- |
| 100 | 50 | 2.59 | 4.7×10^11^ |
| 100 | 100 | 0.79 | 2.9×10^11^ |
| 100 | 200 | 0.81 | 5.0×10^11^ |
| 100 | 300 | 0.64 | 5.2×10^11^ |
| 100 | 400 | 0.57 | 4.5×10^11^ |
| 200 | 50 | 0.63 | 7.8×10^10^ |
| 200 | 100 | 0.43 | 1.1×10^11^ |
| 200 | 200 | 0.38 | 1.9×10^11^ |
| 200 | 300 | 0.14 | 9.6×10^10^ |
| 200 | 400 | 0.23 | 1.9×10^11^ |
| 400 | 50 | 0.06 | 4.0×10^10^ |
| 400 | 200 | 0.10 | 7.6×10^10^ |
| 400 | 200#2 | 0.14 | 8.0×10^10^ |

As the ZnO thickness increases from 100 nm to 400 nm, the device responsivity decreases significantly. According to the analysis in the literature^[5]^, this is because the thickness may exceed the carrier diffusion length in the ZnO layer. The diffusion length is obtained using the following equation:

$$\begin{aligned} L=\sqrt{D\tau}=\sqrt{\frac{k_{0}T}{q}\mu\tau}\#\left( 18 \right) \end{aligned}$$

Here, D, k_0_, q, T, μ, τ represent the diffusion coefficient, boltzmann constant, elementary charge, mobility, and carrier lifetime, respectively. The mobility of the polycrystalline ZnO layer herein is not very high. The carrier lifetime is referenced to the literature^[6]^.

**
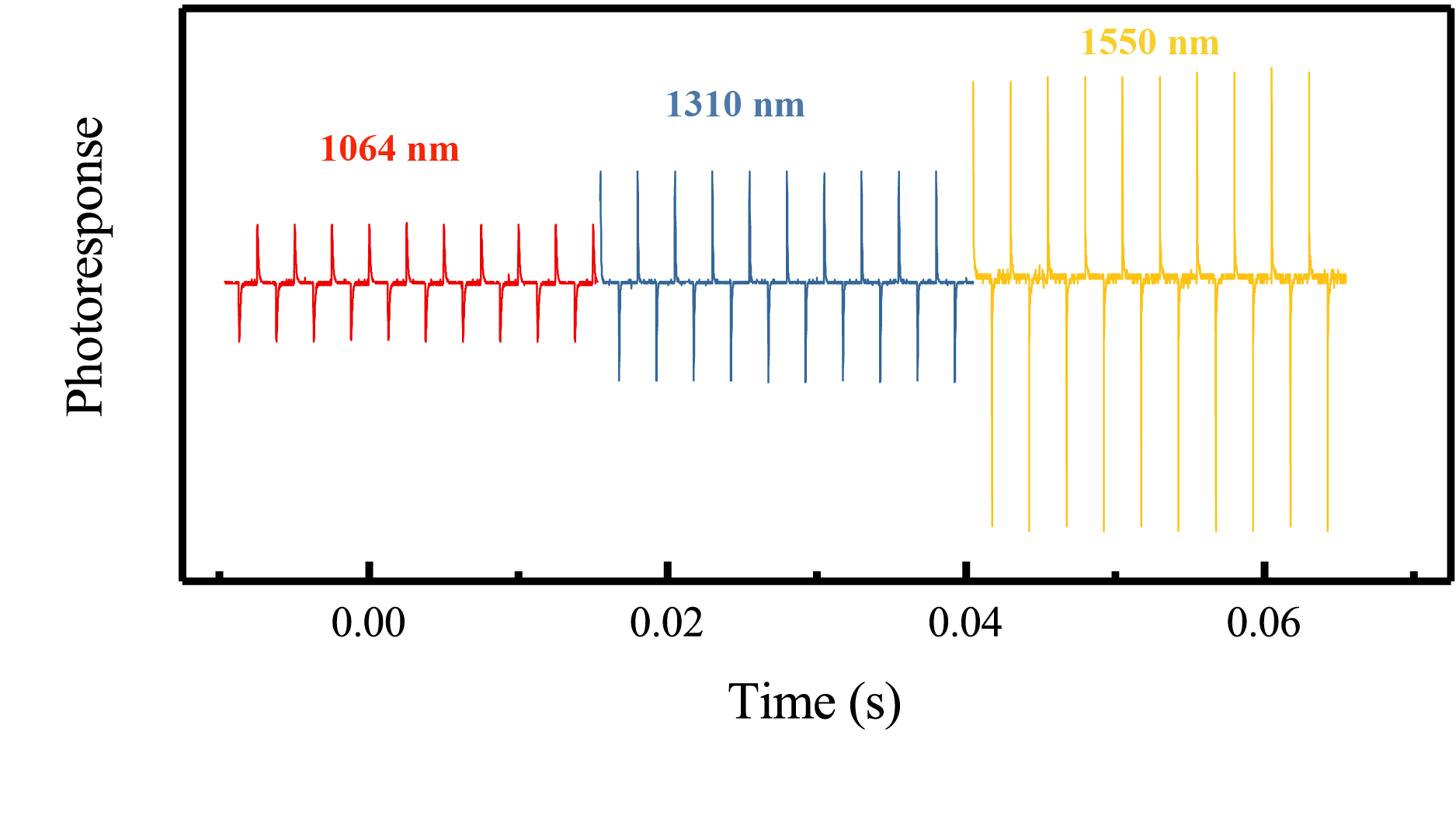
**

**Figure. S14:** Waveforms of the ZnO/PbSe detector under near-infrared irradiation at different wavelengths.

**
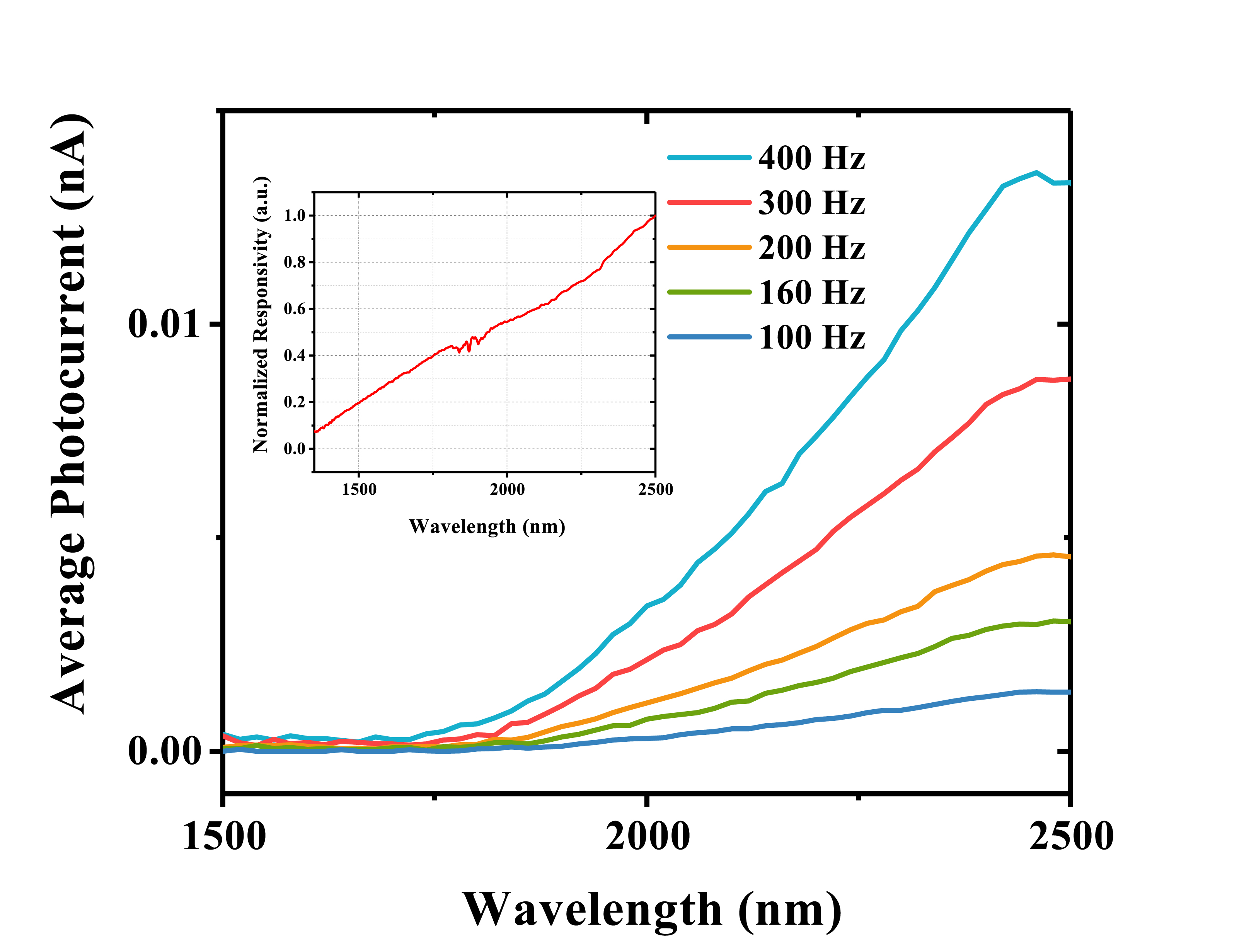
**

**Figure. S15:** Near-infrared photocurrent spectra of the ZnO/PbSe heterojunction devices with different modulation frequencies, the inset shows the near-infrared response spectra of the ZnO/PbSe heterojunction.


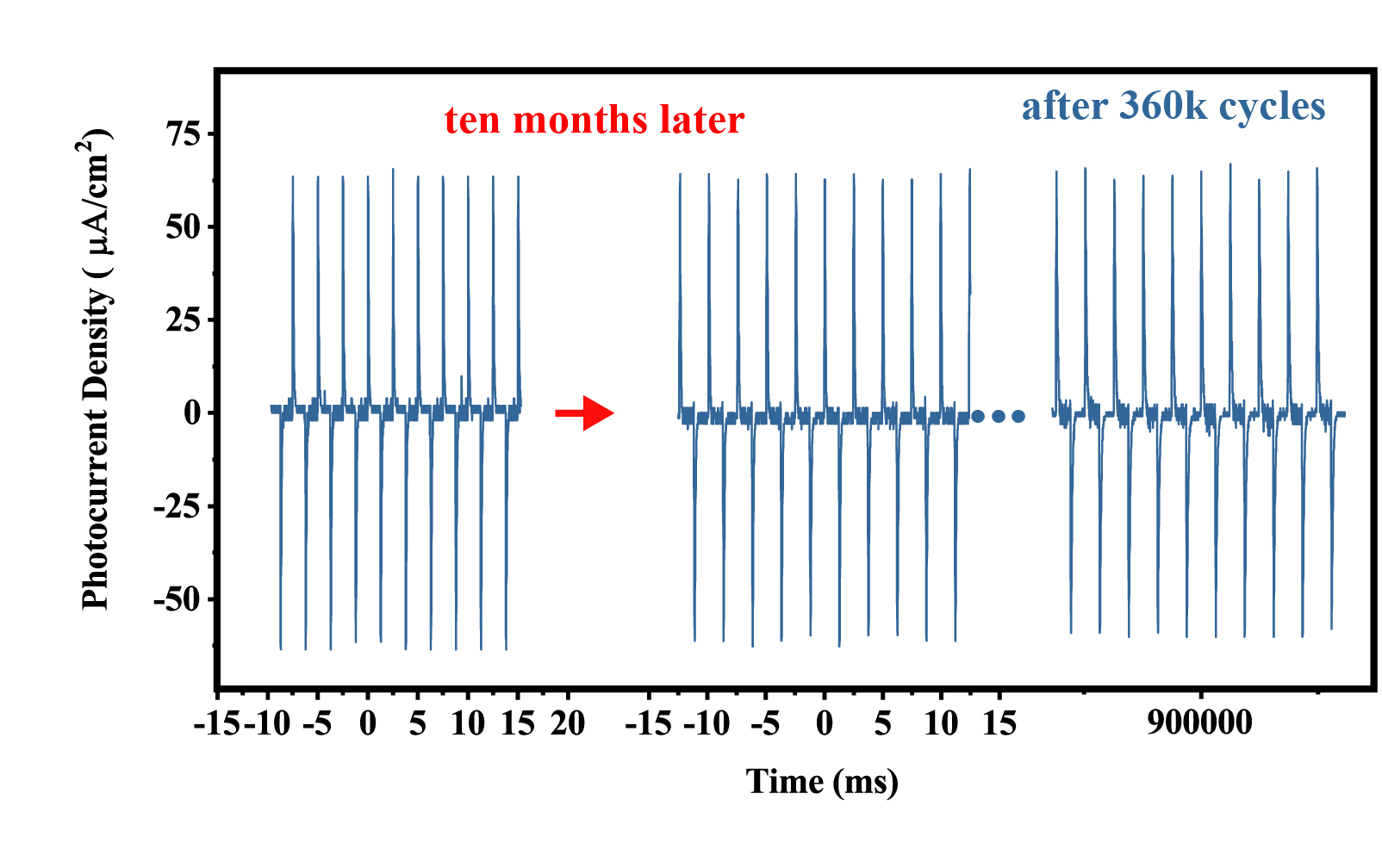


**Figure S16:** Stability test of the device after being stored in the ambient environment for ten months.


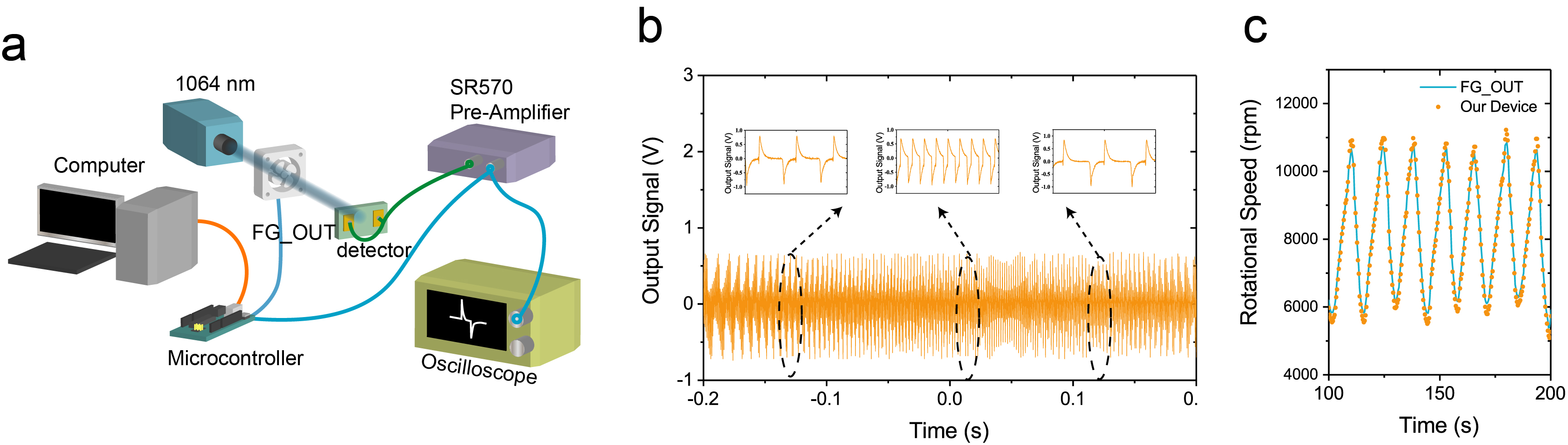


**Figure S17**: (a) Experimental setup for the fan speed measurement. (b) The output waveforms recorded by an oscilloscope; the inset shows the detailed waveforms at different frequencies. (c) Periodic variation of the fan speed measured by our DPD device (yellow dots), in comparison with the motor speed measured by a built-in sensor as a reference (cyan line).

Figure S18(a) shows the speed measurement setup for a frequency-conversion fan as the second application. The recorded signals by an oscilloscope show that the fan speed is continuously changing, as shown in Figure S18(b). By calculating the time interval between adjacent pulses of the detector output, we can obtain the rotational speed of the fan in real-time, as shown in Figure S18(c). Besides, we compare it with the data recorded by a built-in speed sensor of the fan (FG_OUT). From the achieved results, we can see that our DPD detector has great potential to be used in real-time and contactless damage monitoring of high-speed engines without complicated circuits.


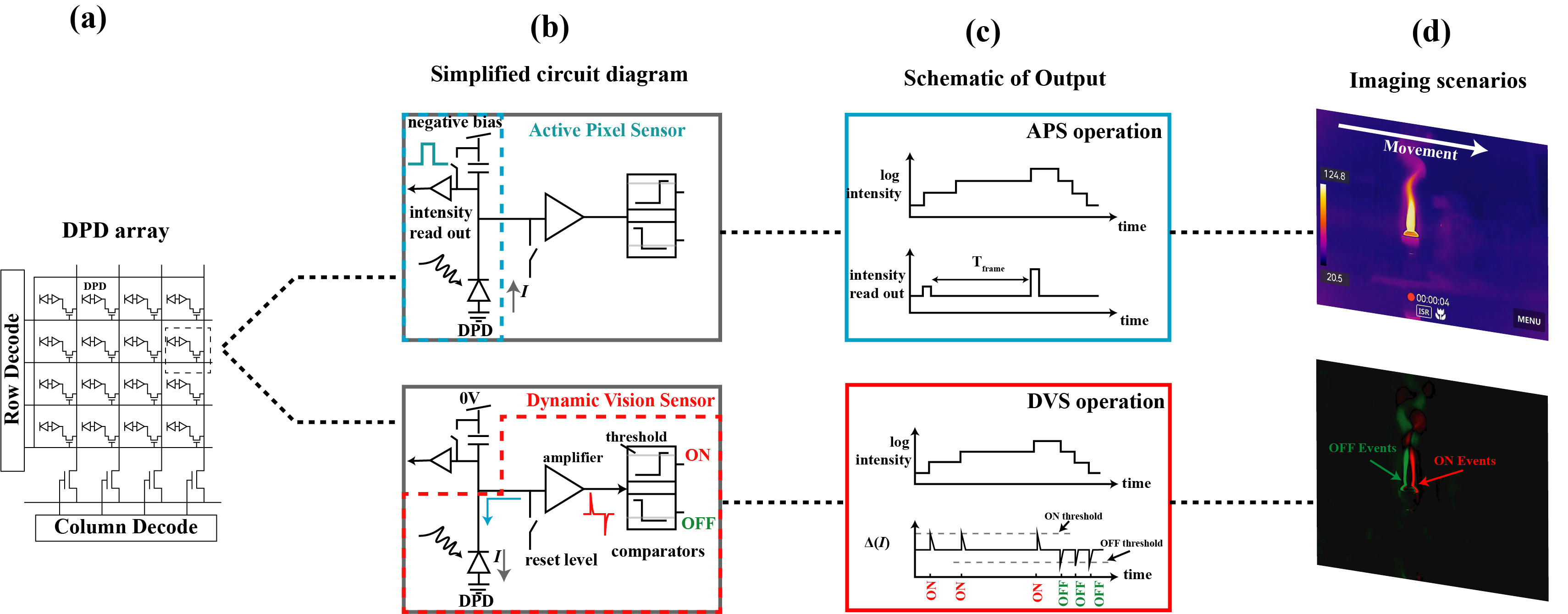


**Figure S18**: Principle of event cameras composed of our devices with two tunable operation modes. (a) Schematic of DPD array with CMOS readout circuits. (b) Simplified circuit diagram of one pixel in the DPD array. Each pixel consists of two sensors: Active Pixel Sensor (APS, corresponding to “normal mode”) and Dynamic Vision Sensor (DVS, corresponding to “differential mode”), while they share the same DPD. (c) Schematic of the output vs time under different operation modes. The output of APS is specified by an external clock (30 or 60 fps). Different from APS, the output of DVS is a data-rate sequence of “events”, where each event represents a change in brightness. Therefore, DVS can achieve high temporal resolution and low power consumption^[7]^. (d) The hypothetical vision under different operation modes.

**Supplementary References:**

[1] B. L. Sharma, R. K. Purohit, “*Semiconductor Heterojunctions,*” Pergamon Press, Oxford, **1974**.

[2] Michael. Shur, “*Physics of Semiconductor Devices / Michael Shur.,*” Prentice Hall, Englewood Cliffs, NJ, **1990**.

[3] In “*Physics of Semiconductor Devices,*” **2006**, pp. 5–75.

[4] J. G. Labram, “Operating Principles of Zero-Bias Retinomorphic Sensors,” *Journal of Physics D: Applied Physics* **2023**, *56*, 065105.

[5] A. Rogalski, J. Rutkowski, “Effect of Structure on the Quantum Efficiency and R0A Product of Lead-Tin Chalcogenide Photodiodes,” *Infrared Physics* **1982**, *22*, 199.

[6] N. Zhou, B. C. Hu, Q. Y. Zhang, C. Y. Ma, S. Z. Hao, “Theoretical Model of Excitonic Luminescence and Its Application to the Study of Fine Structure and Exciton Dynamics in ZnO,” *Journal of Applied Physics* **2019**, *126*, 165701.

[7] G. Gallego, T. Delbruck, G. Orchard, et al., “Event-Based Vision: A Survey,” *IEEE Trans. Pattern Anal. Mach. Intell.* **2022**, *44*, 154.
